# Molecular Analysis of the Cold Tolerant Antarctic Nematode, *Panagrolaimus davidi*

**DOI:** 10.1371/journal.pone.0104526

**Published:** 2014-08-06

**Authors:** Michael A. S. Thorne, Hiroshi Kagoshima, Melody S. Clark, Craig J. Marshall, David A. Wharton

**Affiliations:** 1 British Antarctic Survey, Natural Environment Research Council, Cambridge, United Kingdom; 2 Transdisciplinary Research Integration Center, Research Organization of Information and Systems, Tokyo, Japan; 3 National Institute of Genetics, Mishima, Japan; 4 Department of Biochemistry, University of Otago, Dunedin, New Zealand; 5 Department of Zoology, University of Otago, Dunedin, New Zealand; Universidade de São Paulo, Brazil

## Abstract

Isolated and established in culture from the Antarctic in 1988, the nematode *Panagrolaimus davidi* has proven to be an ideal model for the study of adaptation to the cold. Not only is it the best-documented example of an organism surviving intracellular freezing but it is also able to undergo cryoprotective dehydration. As part of an ongoing effort to develop a molecular understanding of this remarkable organism, we have assembled both a transcriptome and a set of genomic scaffolds. We provide an overview of the transcriptome and a survey of genes involved in temperature stress. We also explore, *in silico*, the possibility that *P. davidi* will be susceptible to an environmental RNAi response, important for further functional studies.

## Introduction

*Panagrolaimus davidi* Timm 1971 [1] was first isolated from the McMurdo Sound region of the Antarctic in 1988 and cultured at the University of Otago [2]. Much like its better known temperate cousin, *Caenorhabditis elegans,* it has, over the years since its early adoption as a model of study, provided surprises that in hindsight made its initial selection, and culturing, very fortunate. *Panagrolaimus davidi* was found to survive intracellular freezing [3]. Although the cuticle acts as a barrier to ice, at low sub-zero temperatures inoculative freezing can occur through the excretory pore and other orifices, with the ice seeding the body fluid and freezing both intra- and extracellular compartments [3], [4]. Survival of intracellular freezing was first described by Salt in the fat body cells of the goldenrod gall fly [5], [6], [7] but *P. davidi* still remains the only organism for which it has been described in all compartments of its body. In addition to freezing tolerance, *P. davidi* has developed other strategies for dealing with low temperatures. At high sub-zero temperatures, and importantly, when the rate of freezing is slow, ice is unable to enter body openings and the nematode supercools. This leads to a vapour pressure difference between the supercooled body fluids and the surrounding ice leading to the transfer of water from the nematode to the surrounding ice; a process termed cryoprotective dehydration [8], [9]. Once *P. davidi* is cryoprotectively dehydrated, there is not enough water to freeze in either the pseudocoel or other compartments allowing it to sustain low sub-zero temperatures by freeze avoidance. While an increasingly detailed molecular picture is emerging of cryoprotective dehydration using large scale gene expression and proteomic approaches [10], [11], [12], no comparable molecular work has been undertaken on the survival of intracellular freezing.

With the sea change in current sequencing technology, what was previously termed a non-model organism can now become, with a concerted effort and a fraction of the resources that were required even five years ago, a molecular model for a specific physiological trait, which is a powerful change in the way biological systems can now be studied. With this in mind, we have undertaken to sequence not just the transcriptome, which will provide a backbone for expression studies, but also the genome. With the dramatic decrease in cost associated with sequencing genomes, the scaffolds and contigs resulting from a preliminary assembly, even when broken up, are a natural complement to the transcriptome, providing valuable information on gene structure such as intron-exon junctions. To date our only knowledge of the genome comes from a paper by Goldstein and Wharton [13] describing seven synaptonemal complexes, therefore seven chromosome pairs (2n = 14).

The molecular information provided on the scale such as the present study, opens up the exploratory possibilities of gene expression studies. But in order to gain more direct evidence for any such exploratory results, it would be valuable to know whether *P. davidi* is potentially susceptible to functional genetic methodologies. Since Fire *et al.* [14] determined the role of RNA interference in *C. elegans*, this method has provided a clear mechanism by which the role of a pathway or specific genes may be understood, for example, in an organism’s response to environmental stresses [15]. With the two sources of information, the transcripts and their gene structure determined by the genomic sequence, clean RNAi probes for specific targets can be easily developed. Recently, *Panagrolaimus superbus* was shown to respond to feeding RNAi [16], but a lesson has already been learnt from *C. briggsae* when, unlike *C. elegans* for whom it has been a comparative model, it was shown that RNAi was not possible owing to a divergent form of *sid-2* [17].

A final word on a mystery that has unfolded in the last few years. In 2009, Lewis *et al*. published a paper [18] on the phylogenetics of the *Panagrolaimus* genus. *P. davidi* (designated CB1, from its presumed origin at Cape Bird, Ross Island) was included in this study, but unexpectedly, two Californian species proved phylogenetically closer to *P. davidi* CB1 than any other species or strain. Genetic analysis of fresh field material of *P. davidi* collected during 2005–2007 from Cape Hallett and Gondwana Station on the Victoria Land coast and from Cape Bird showed that the field strain of *P. davidi* is a different species to *P. davidi* CB1 [19]. One argument put forward to explain this difference is the possible dominance in culture of a less common strain owing to its parthenogenetic reproductive mode. Questions of invasiveness have also been considered. But the adaptations of *P. davidi* CB1 to low temperature make this highly unlikely; or highly surprising. Further molecular work should help to resolve this intriguing situation, but in terms of its physiology, the origin of *P. davidi* CB1 is not of relevance, since its cold tolerance adaptations singles it out as an important organism of study.

## Materials and Methods

### Culturing, extraction, and library construction for Expressed Sequence Tags

Nematodes were cultured on *Escherichia coli* strain OP50 on NGM agar plates [20]. RNA was extracted from 580 mg (for a culture grown at 20°C, called PDT) and 730 mg (for a culture grown at 20°C and subsequently brought down to 4°C, called PDF) of *P. davidi* CB1 (wet weights), respectively. Total RNA (930 µg for PDT and 750 µg for PDF) was prepared with RNagents total RNA Isolation Kit (Promega). Poly(A)+ RNA (2.9 µg for PDT and 5.5 µg for PDF) were isolated with Illustra mRNA Purification Kit (GE healthcare). 2 µg of poly(A)+ RNA was then used to generate a cDNA library with Cloneminer cDNA library Construction Kit (GE healthcare).

### Sanger sequencing and quality assurance

Seqclean [21] and Crossmatch [22] was applied to the two (PDT and PDF) *P. davidi* CB1 Expressed Sequence Tag (EST) libraries, removing the vector and stripping any poly-A tails and poor quality sequence. This left 25,182 reads from the PDT set and 69,958 reads from the PDF set. All of these sequences were added to the post-assembly of the Illumina reads. The ESTs are held in dbEST with accession numbers: JZ585947–JZ681086.

### Culturing, extraction, and library construction for Illumina sequencing

The nematodes were cultured in S medium at 20°C for 3 weeks and fed every 3–4 days with *E. coli* following [23]. Nematodes were extracted using a modified Baermann technique [24]. The worms for the DNA and RNA were then snap frozen with liquid nitrogen in 1.5 ml microcentrifuge tubes and preserved at −80°C until extraction. Seven sets of these worms for the RNA were then subjected to other physiological states to enrich for stress related transcripts. These seven treatments consisted of 1) exposure to cold acclimation at +5°C for 3 days*;* 2) the previous sample set was then immersed in the bath of a refrigerated circulator where it was cooled from +1°C to −1°C at 0.5°C min^−1^, and frozen by adding a small ice crystal and maintained at −1°C for 24 h; 4) the previous sample was warmed to +1°C at 0.5°C min^−1^, and allowed to recover at 20°C for 24 hours; 5) samples from stage 1 were cooled from +1°C to −10°C at 0.5°C min^−1^; 6) the previous stage was ice nucleated once held at −10°C; 7) the previous sample was warmed to +1°C at 0.5°C min^−1^. 500 ng (wet weight) was used for the RNA extraction (from each of the 8 different stages, including the culture grown at 20°C) and 500 ng (wet weight) for DNA extraction. The RNA was extracted from whole worms using TRI-sure (Bioline) according to manufacturer’s instructions and purified on Qiagen RNeasy columns. The DNA was extracted from whole worms using the Qiagen DNAeasy Blood and Tissue kit according to manufacturer’s instructions. Both DNA and RNA were checked for purity on standard agarose gels and quantified using a NanoDrop ND-1000 spectrophotometer (Labtech). 8 µg of DNA and 10–15 µg of RNA for each of the stages were used for sequencing.

### Illumina sequencing and quality assurance

The RNA was sequenced on an Illumina HiSeq 2000 resulting in 143,223,606 paired-end reads of 100 bp, after quality control. Quality control consisted of removing adaptors from the sequence, removing reads where the number of unresolved nucleotides exceeded 5%, and reads where the number of nucleotides with phred quality less than or equal to 10 was over 20%. The reads can be downloaded from the SRA repository under the accession number SRP041973.

Genomic DNA was randomly fragmented, with insert sizes of between 500–800 selected through gel electrophoresis, with the fragments gel purified with adapters ligated. After sequencing, quality control consisted of adaptor removal and removing reads in which more than 50% of the bases had phred scores of less than 5. Two runs of 95 and 138 million paired end reads of 100 bp respectively remained after quality control. The sequence data can be downloaded from the SRA repository under the accession number SRP041572.

### Transcriptome assembly and annotation

The transcriptome was assembled from 143,223,606 paired-end Illumina reads of 100 bp, and 95,140 sanger sequences. The Illumina reads were assembled first with Soapdenovo [25] using a number of different kmer sizes. Illumina datasets assemble differently depending on the kmer size chosen, with any given dataset having an optimal kmer for numbers and lengths of contigs. In the case of *P. davidi* CB1, by selecting a number of odd values from 19 to 93, the optimal kmer lay around 69 (see File S1). In order to enrich the assembly as much as possible, all contigs greater than 200 bp from all the different kmer size assemblies were selected (a total of 1,107,215 contigs) and, jointly with the 95,140 sanger sequences, they were assembled together using Newbler [26] and CAP3 [27] consecutively, to eliminate redundancy. The resulting assembly was compared using Blast [28] against the nr database [29] as well as *Caenorhabditis elegans* [30], *Plectus murrayi* [31] and *Panagrolaimus superbus* [32] nematode databases. In addition, a separate blast against the entire WS242 wormbase [30] was carried out. The final assembly set was reduced to those transcripts that were either 500 bp long, or annotated at an e-value less than 1e-10.

Functional groupings of the transcriptome was carried out through Clusters of Orthologous Genes [33], Kegg Orthology [34], and SEED subsystems [35]. Separate annotation of the ESTs was also carried out and they were used in examining specific genes, particularly the LEA-like and HSP-70 genes, owing to their longer lengths combined with their more easily resolved reading frame, where the consensus sequences in the contigs proved more complicated.

### DAPI staining of nuclear DNA

*C. elegans* N2 and *P. davidi* CB1 were washed out from a 5 cm Nematode Growth Media (NGM) plate and fixed in 2 ml Carnoy’s solution (60% Ethanol, 30% Chloroform, 10% Acetic acid) overnight at room temperature. The fixed worms were then transferred to a watch glass with 50 µl phosphate buffered saline with 0.2% Triton X-100 and 100 ng/ml DAPI (4′,6-diamidino-2-phenylindole), and incubated in a humid chamber in the dark for 30 mins. The worms were mounted on an agar pad slide. Quantification of the intensity of nuclei in ventral nerve cord was carried out with AQUACOSMOS software [36] on fluorescent microscopy pictures with an Axioplan 2 microscope (Carl Zeiss). Estimation of *P. davidi* CB1 DNA amounts by proportion to *C. elegans* DNA was done for 10 worms from each species.

### Assembling genomic sequence

As with the transcriptome assembly, Soapdenovo, with varying kmer sizes, was used to assemble the DNA paired-end reads. This led to an optimal kmer size of 89. Since it has often been noted that too much sequence coverage can lead to erroneous and more broken assemblies, a second, confirmatory round of assemblies were carried out with only 50x coverage, and then at 50x increments, to see whether the assembly at any lower coverage was better. However, this resulted in confirmation that the more data, the better the assembly. Finally, Blast was used to check and remove any contigs or scaffolds that may have been an assembly of bacterial contaminant.

## Results and Discussion

The transcriptome, consisting of 25,875 transcripts, had an average length of 1,163 bp with the GC content at 32.5% (the raw Illumina reads were 35.97%). Against the nr database, 15,748 (61%) of the transcripts were annotated at an e-value of 1e-10 or less. When compared to the *C. elegans* protein database using blastx, 14,372 (54%) *P. davidi* CB1 *(*hereafter referred to simply as *P. davidi)* transcripts matched 14,395 (57%) of the *C. elegans* proteins. The *P. davidi* transcripts matched 62% of the *Panagrolaimus superbus* EST transcripts [32], the closest nematode for which there is any molecular data, and they matched 52% of the *Plectus murrayi* ESTs [31], the only other Antarctic nematode for which any molecular data has been published. When compared to all nematode databases currently housed at wormbase (WS242), 68.5% of the *P. davidi* transcripts matched at an e-value of 1e-10 or lower. The remaining transcripts that had no annotation, and in particular, those that matched no nematode are obviously of interest in that they may well contain potential clues to the unique physiological adaptations of *P. davidi.* File S2 lists all the transcripts that match against nr at an e-value of 1e-10 or lower, along with their corresponding description.

**Figure 1:**
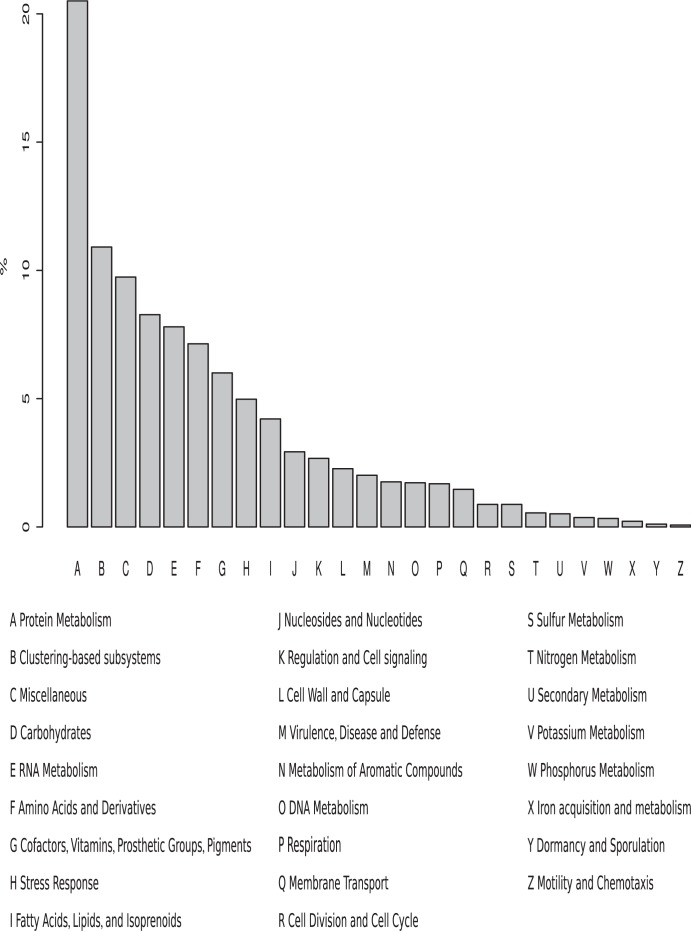
SEED subsystem analyses of the transcriptome. The y-axis indicates the percentage of the total annotated set represented by the specific category.

Functional classifications were able to be determined for 55% of the whole transcriptome. Figure 1 shows the functional spread provided by SEED subsystems. Although not shown, both the Clusters of Orthologous Genes and KEGG Orthology analyses, while broader in their categories, provided the same breakdown. The highest proportion of the transcriptome is clearly involved in protein metabolism, with a high proportion of clustering-based subsystems, a designation of genes based on their co-localisation across many genomes. Other highly represented subsystem groups were the carbohydrates, amino acids and derivatives, and RNA metabolism. A separate analysis was done on the two EST libraries with the breakdowns showing similar percentage patterns (File S3) with, like the transcriptome as a whole, protein metabolism having the strongest representation.

That the transcriptome is characterised so strongly by genes involved in protein turnover is a clear indication of activity and change which is hardly surprising given the fact that different physiological states were mixed together, where such activity might be expected. With the similar proportions also apparent in the two EST libraries this seems to imply that such activity and change is occuring even within each stage. This is probably reflective of the need to be highly responsive to changes in the environmental conditions.

A natural complement to the transcriptome, even in preliminary form, is the genome sequence. Prior to sequencing however, it is useful to know the size of the genome, even approximately. DAPI staining and comparison to the *C. elegans* nuclear DNA was carried out with the result that *P. davidi* is estimated to be roughly ∼90 Mb, slightly smaller than *C. elegans* at ∼97 Mb. We then sequenced two short-insert paired-end libraries (<1,000 bp) of nuclear DNA, which according to the genome size by DAPI staining, would provide a sequence depth of coverage of roughly 517. Assembly resulted in 86% inclusion of the incorporated genomic reads. The scaffold N50 is 6,352 bp, with an average size of 4,150 bp, and a total size of 195 Mb. File S4 shows the size distribution of the scaffolds greater than the N50 with the largest at 73,240 bp (the N50 of the contigs (those joined together to form the scaffolds) is 1,873 bp with the largest being 21,613 bp with the total size being 93 Mb).

The total size of the scaffolds indicates an observation that had already been noted with the EST libraries, namely that while *P. davidi* is a parthenogenetic species, which should give rise to a homozygous line, evidence suggests that it is in fact heterozygous. Generation of the ESTs was constructed from a single animal with >10x generations to produce an isogenic line, yet many of the ESTs showed a heterozygosity. Examining the LEA ESTs for example, provided evidence that is best explained by heterozygous descendent sequences in which there is crossing over. This was followed up by examining the LEA loci on the genome, as well as examples in the cDNA of two other genes, glutamine synthetase and 6-phosphogluconate dehydrogenase, all of which led to the same conclusion (see File S5).

**Figure 2:**
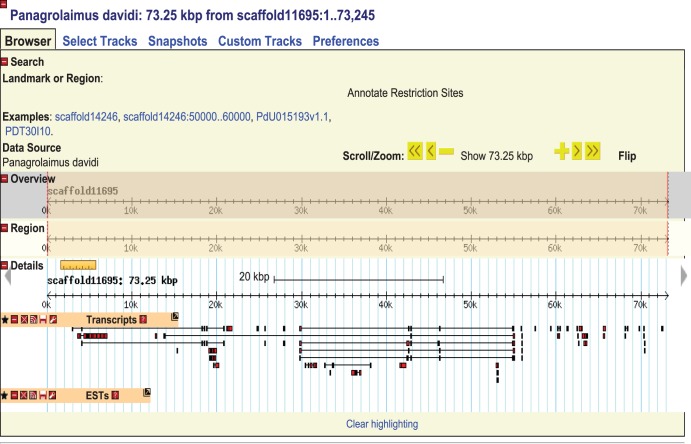
Browser of the scaffolds and their transcript alignments, housed at genomes.bas.ac.uk. The browser will be updated during the continued development of the genome assembly.

Mapping of the transcriptome onto the scaffolds resulted in 94% of the transcripts finding a match at an e-value of 1e-10 or lower, indicating that while broken up, the genome sequence is reasonably complete. Further work is being carried out to build the assembly into a more contiguous form (see Figure 2). Subsequent work on building up a well-annotated genome should also be made easier with close comparative models, such as other *Panagrolaimus* species.

The genomic scaffolds that have been assembled have already provided valuable information for the development of PCR probes, allowing one to examine the splice sites for many of the transcripts, as well as for current work in developing probes for RNAi. A small example resulting from the assembly that is redolent of the early work on the *C. elegans* genome providing information on gene structure is the intron size that was found to have a peak at 47 bp [37], and in *C. briggsae* at 54 bp [38]. For *P. davidi*, 139 random intronic regions were examined in closer detail across 37 scaffolds showing a clear peak at 49 bp (see File S6).

One of the key reasons for deep sequencing and undertaking a large-scale genomics approach in *P davidi*, is to aid in isolating key genes involved in cold tolerance. One class of protein that has proven elusive so far, despite a previously reported but inconclusive result [39] are the ice-active proteins (IAP): ice nucleating proteins (INPs), antifreeze proteins (AFPs) and recrystallization-inhibiting proteins (RIPs) [39]. Adhikari *et al.* [31] has reported a type II antifreeze protein in *Plectus murrayi*, and it was expected that such a find would also be made in the current *P. davidi* transcript set. However, neither searches of the annotation or homology matches with the *Plectus murrayi* EST has identified such a gene, except for a very weak match to transcript PdU008960v1.1, a c-type lectin carbohydrate-binding protein. To date all effort to find even one ice-active protein has failed. In 2009, a newly discovered antifreeze molecule, xylomannan, was isolated from the freeze tolerant Alaskan Beetle, *Upis ceramboides* [40]. Since this is not a protein, but a combination of saccharide and fatty acid, such an avenue may prove useful with *P. davidi*, but has yet to be undertaken.

Much is already known of the molecular processes involved in cryoprotective dehydration [10], [11], [41] and Table 1 lists a number of the key genes which have been identified in previous expression profiling studies. These include genes in the trehalose synthesis pathway, the aquaporins, chaperones and oxidoreductase genes associated with cell stress, and desaturase genes involved in membrane fluidity.

**Table 1:** Associated *P. davidi* transcripts matching genes significant for cold tolerance.

Function	*C elegans* homolog	*P davidi* contig	E-value
Trehalose -6-phosphate synthase	tps-2	PdU054346v1.1	0
	tps-2	PdU054198v1.1	e-133
Trehalase	tre1	PdU000858v1.1	0
	tre-2	PdU000867v1.1	0
	tre-3 isoform a	PdU002260v1.1	e-165
	tre-3 isoform b	PdU001362v1.1	e-165
Trehalose -6-phosphate phosphatase	gob-1	PdU001633v1.1	e-145
Aquaporin	aqp-2	PdU054401v1.1	e-067
	aqp-3	PdU008003v1.1	e-082
	aqp-4	PdU055737v1.1	e-059
	aqp-7	PdU008968v1.1	e-89
	aqp-7	PdU011262v1.1	e-99
	aqp-8	PdU054717v1.1	e-076
Desaturase	fat-2	PdU004098v1.1	e-136
	fat-7	PdU004305v1.1	e-116
	fat-7	PdU004197v1.1	e-115
Superoxide dismutase	sod-1	PdU057258v1.1	e-051
Catalase	ctl-1	PdU002908v1.1	0
Peroxyredoxin	prdx-2	PdU058248v1.1	e-090
Glutathione peroxidase	gpx-1	PdU020041v1.1	e-060
Glutaredoxin	glrx-5	PdU019570v1.1	e-040
Glutathione S-transferase	gsto-1	PdU008618v1.1	e-037
	gst-21	PdU016688v1.1	e-030
	gst-1	PdU015378v1.1	e-022

Trehalose is commonly synthesised from glycogen and has been shown to act as an anhydroprotectant [42] by preserving the functionality of biomolecules and acting as a water replacement in terms of a compatible osmolyte [43], and by glass formation and chemical stability [44]. Two enzymes are directly involved in the synthesis of trehalose: trehalose-6-phosphate synthase (tps) and trehalose 6-phosphate phosphatase (gob), with trehalase (tre) involved in the breakdown of this sugar. Previous work on desiccation has identified a duplication of the tps gene in other species: *Megaphorura arctica* [11], *C. elegans* [45] and *Brachionus plicatilis* [46]. Transcripts with sequence similarity to trehalose-6-phosphate synthase (*tps*) were identified in the *P. davidi* dataset, but these appeared to be two non-overlapping portions of the same gene, namely *tps-2*. However searching the genomic scaffolds indicate that they may come from different regions of the genome, and therefore a potential duplication. *TPS-1* was not identified in this dataset. However, in line with the previous studies on duplicated *tps* genes, potential duplicates of the trehalase gene were also identified in *P. davidi*. Homologs of the remaining two tre genes, *tre-4* and −5 that are present in *C. elegans*, were not found.

Two membrane function gene families have been identified as being significantly expressed during cryoprotective dehydration, the aquaporins (associated with solute transport across membranes) [47] and the Δ9-acyl-CoA desaturases (involved in changing membrane fluidity via fatty acid composition) (eg [48]). To date 12 aquaporin (*aqp*) and 7 desaturase (*fat*) genes have been identified in the genome of *C. elegans*. In the *P. davidi* dataset 6 *aqp* and 3 *fat* genes were identified. Of particular note was the potential duplication of both *aqp-7* and *fat-7*, which would be specific to *P. davidi*. Searching the genomic scaffolds, both *fat*-7 transcripts aligned onto the same scaffold implying different regions of the same gene. However, the two transcripts of *aqp-7* aligned to different scaffolds, which indicate a potential duplication. It is worth noting that *aqp-7*, as well as *aqp-3*, are the aquaglyceroporin genes, the glycerol-permeable homologs of the classical aquaporins for water transport.

Much of the recent molecular interest in desiccation survival has involved the study of the late embryogenesis abundant (LEA) protein family. These were initially found during the embryogenesis of cottonseed in 1981 [49], [50] and are hydrophilic, intrinsically disordered proteins. They have received a great deal of attention over the last decade or so, since they were found to play a role not only in the desiccation of plants, but also in animals. The verdict is still out in terms of both the classification system that should define the types – the plant types do not so easily translate to the animal types – but also in terms of all the possible functions the LEAs might play [51]. This paper will not attempt to weigh into the debate on the types, as there has been some good work to date focussed on this issue [52], [53], [54], [55]. Owing to the inconclusive designations many researchers prefer to refer to certain LEAs found in animals as LEA-like (i.e. [56]) with all LEA-related proteins found in animals to date most similar to type 3 LEA, with the exception of two type 1 LEA sequences from *Artemia franciscana* [57]. More work also needs to be done on differentiating the functional differences of the separate types before too much is made of the syntax, even though it is likely that any syntactic differences will be reflected functionally. As Tunnacliffe and Wise [51] have phrased it, the LEAs remain a conundrum.

**Figure 3:**
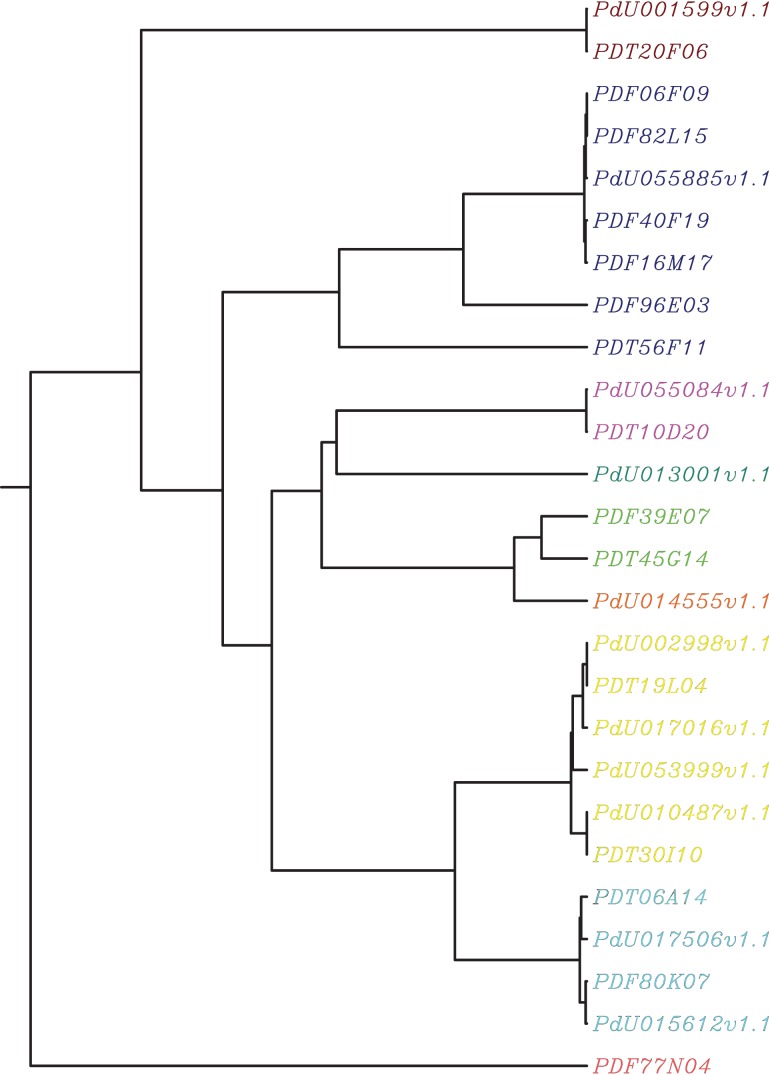
UPGMA clustering of the LEA transcripts and ESTs from **Table 2**. The colouring represents those transcripts and ESTs that aligned on the same scaffolds, as shown in Table 2. The close clustering and independent scaffold alignment provides evidence of distinct LEA genes.

Among the many functional properties attributed to the LEA (and LEA-like) proteins are as a molecular shield inhibiting aggregation of denaturing proteins, as an antioxidant, to provide protection to membranes preventing damaging phase transitions during freezing, as hydration buffers, slowing water loss - among others (see [51], [58], [56]). In nematodes trehalose is an important constituent of desiccation survival [59], unlike in tardigrades and bdelloid rotifers where trehalose is not accumulated, or even present during desiccation [60], [61], [62]. Yet Gal *et al.* [63] found that silencing the *C. elegans lea-1* gene significantly reduced survival during induction of desiccation as well as of osmotic and heat stress. However, the more remarkable of recent studies is one conducted on human hepatoma cell lines in which two type 3 LEA proteins from *Artemia fransiscana* were transfected, with 98% of cells retaining membrane integrity after rehydration from low water content, compared to 0% without transfection [64]. When the same was attempted without the trehalose and only one of the transfected LEAs, 94% of the cells retained membrane integrity. Within the current *P. davidi* dataset, both the contigs and the EST reads were searched for potential LEAs with at least 26 individual reads or contigs found. These were checked for the ability to compose an amphiphilic α-motif [65], [51], and whether they were natively unfolded using Foldit [66]. These ESTs and contigs were then clustered using Clustal [67] (see Figure 3) as well as checked for homology to the genomic scaffolds (see Table 2). The colouring scheme found in Figure 3 depict those sequences found on the same scaffold (in Table 2). As can be seen, the colouring matches perfectly the independent clustering based on Clustal which provides evidence that there are possibly up to 9 different LEA-type genes. However, translation to amino acid, combined with Cd-hit [68] at a relatively low stringency threshold of 0.8, resulted in 13 separate clusters. File S7 contains the cd-hit clustering and the resulting representative sequences.

**Table 2:** *P. davidi* LEA-like sequences.

Transcript/EST	Length (aa)	Unfoldability*	Charge*	Phobic*	Genomic Scaffold‡
PdU001599v1.1	339	−0.078	0.006	0.388	n/a
PDT20F06	594	−0.023	0.015	0.41	n/a
PDF06F09	288	−0.189	0.031	0.356	Scaffold19015
PDF82L15	201	−0.18	0.06	0.37	Scaffold19015
PdU055885v1.1	307	−0.146	0.023	0.369	Scaffold19015
PDF40F19	317	−0.189	0.028	0.356	Scaffold19015
PDF16M17	306	−0.194	0.029	0.354	Scaffold19015
PDF96E03	209	−0.225	0.115	0.374	Scaffold19015
PDT56F11	172	−0.128	0.029	0.378	Scaffold19015
PdU055084v1.1	235	−0.203	0.047	0.357	Scaffold51708
PDT10D20	235	−0.206	0.047	0.356	Scaffold51708
PdU013001v1.1	169	−0.109	0.065	0.398	Scaffold10457
PDF39E07	90	−0.076	0.022	0.394	Scaffold18176
PDT45G14	98	−0.084	0.041	0.398	Scaffold18176
PdU014555v1.1	150	−0.049	0.033	0.408	Scaffold1422
PdU002998v1.1	502	−0.181	0.008	0.351	Scaffold12112
PDT19L04	1002	−0.151	0.009	0.362	Scaffold12112
PdU017016v1.1	200	−0.179	0	0.349	Scaffold12112
PdU053999v1.1	593	−0.148	0.007	0.363	Scaffold12112
PdU010487v1.1	272	−0.171	0.011	0.356	Scaffold12112
PDT30I10	593	−0.129	0.002	0.368	Scaffold12112
PDT06A14	320	−0.06	0.034	0.404	Scaffold38706
PdU017506v1.1	119	−0.175	0.05	0.369	Scaffold38706
PDF80K07	282	−0.049	0.032	0.407	Scaffold38706
PdU015612v1.1	234	−0.076	0.017	0.392	Scaffold38706
PDF77N04	78	−0.359	0.103	0.321	Scaffold59957

*see [66].

‡ Independent clustering of transcript similarity can be seen in Figure 3.

Although too many to be included in Table 1, another important class of temperature stress proteins involved in protein stability are the numerous heat shock proteins. *C. elegans* has 12 types of HSP-70 genes, with two pairs (F44E5.4/F44E5.5 and HSP-3/4) almost identical, suggesting they have been raised by gene duplication. In *P. davidi,* analysis to date has indicated 7 HSP-70 homologs, with 20 HSP-70-like genes. The *P. davidi* sequence provides homologs of HSP-1, HSP-3/4, HSP-6, HSP-70, HSP-110, F44E5.4/.5, and T14G8.3, with HSP-1, HSP-3/4 and HSP-6 indicating the presence of orthologs. File S8 provides the *P. davidi* sequences of the different groups.

For the cold tolerant process of intracellular freezing, very little is as yet known of the mechanisms that allow this to occur, with no molecular work done on any organism to date. However it would be surprising if many of the above mentioned genes were not in some way involved, either mechanistically, or in terms of stress response.

From the beginning of the molecular focus on *P. davidi*, a vital question has been whether it is susceptible to environmental RNA interference. If so, it would provide a method of functionally investigating survival of intracellular freezing. We have provided an *in silico* search for RNAi specific genes (following [69]). As with the IAP, an inability to find a key gene does not preclude there being one. However, so far there has been no *in silico* evidence of *sid-2*, even though a number of other associated genes are present (see Table 3). As pointed out in the introduction, without *sid-2*, even if other associated RNAi genes were present, it is considered unlikely that *P. davidi* would have an environmental response to RNAi [17]. The results could potentially mirror the relationship between *C. elegans* and *C. briggsae,* since *Panagrolaimus superbus* has been shown to have an RNAi response [16]. Work in determining whether there is an environmental response is being done in Otago, but so far the results have been inconclusive (A. Seybold, per. comm.). If the information hinted at in the *in silico* search is correct, it would be disappointing, since any lack of response would imply similar difficulties with the soaking method [70]. While microinjection is still a possibility in providing an RNAi response [14], it is unlikely to be of help in understanding an environmental response where large numbers of nematodes are needed to provide a statistical indication of survival.

**Table 3:** An *in silico* search of the *P. davidi* transcriptome for the genes associated with RNAi (following [69]).

Function	*C elegans* homologs	*P davidi* contigs	E-value*
Small RNA biosynthetic proteins	drh-3	PdU000268v1.1	e-83
	drsh-1	PdU006095v1.1	e-73
	xpo-1	PdU054092v1.1	0
	xpo-2	PdU003765v1.1	e-31
	dcr-1	PdU000401v1.1	e-139
	drh-1	PdU000369v1.1	e-63
	pash-1	PdU009300v1.1	e-16
	rde-4	PdU000474v1.1	e-47
	xpo-3	-	
dsRNA uptake and spreading,and siRNA amplification effectors	smg-2	PdU000228v1.1	0
	smg-6	-	
	ego-1	PdU000148v1.1	e-177
	rrf-3	PdU000148v1.1	e-122
	rrf-1	PdU000148v1.1	e-175
	smg-5	-	
	rsd-2	-	
	rsd-3	PdU001889v1.1	e-39
	sid-1	-	
	rsd-6	-	
	sid-2	-	
Argonautes (AGOs) andRNA-induced SilencingComplex (RISC) components	alg-1	PdU000474v1.1	0
	R06C7.1 (wago-1)	PdU000507v1.1	e-102
	C04f12.1	PdU000451v1.1	e-52
	F58G1.1 (wago-4)	PdU000507v1.1	e-105
	alg-4	PdU000474v1.1	e-135
	rde-1	PdU000474v1.1	e-47
	C16C10.3 (wago-9)	PdU055266v1.1	e-54
	ppw-1	PdU000507v1.1	e-53
	csr-1	PdU000451v1.1	e-50
	ppw-2	PdU000507v1.1	e-97
	sago-1	PdU000453v1.1	e-39
	T22B3.2	PdU000474v1.1	e-128
	T22H9.3 (wago-10)	-	
	alg-2	PdU000474v1.1	0
	ergo-1	PdU000474v1.1	e-49
	prg-1	PdU000474v1.1	e-32
	F55A12.1 (wago-2)	PdU000453v1.1	e-72
	T23D8.7 (hpo-24)	PdU000474v1.1	e-170
	nrde-3	PdU055266v1.1	e-38
	sago-2	PdU000507v1.1	e-53
	T23B3.2	-	
	Y49F6A.1 (wago-11)	PdU055266v1.1	e-49
	ZK1248.7 (wago-5)	PdU000507v1.1	e-107
	prg-2	PdU000474v1.1	e-29
	C06A1.4	PdU000507v1.1	e-88
	C14B1.7	PdU055266v1.1	e-46
	tsn-1	PdU002202v1.1	e-131
	ain-1	-	
	vig-1	PdU004590v1.1	e-13
	ain-2	-	
RNAi inhibitors	eri-1	PdU006594v1.1	e-29
	xrn-2	PdU056342v1.1	e-129
	adr-2	PdU002126v1.1	e-24
	xrn-1	PdU056342v1.1	e-64
	adr-1	-	
	lin-15b	-	
	eri-5	-	
	eri-6/7	PdU001476v1.1	e-45
	eri-3	-	
Nuclear RNAi effectors	mut-7	PdU057227v1.1	e-15
	cid-1	PdU001764v1.1	e-26
	ekl-1	PdU010210v1.1	e-12
	gfl-1	PdU010550v1.1	e-32
	mes-2	PdU000971v1.1	e-57
	rha-1	PdU053907v1.1	e-148
	ekl-6	PdU000462v1.1	e-27
	zfp-1	PdU000768v1.1	e-27
	mut-2	PdU001764v1.1	e-22
	ekl-5	-	
	mes-3	-	
	mut-16	-	
	rde-2	-	

*Homologs not found at the e-value cutoff of 1e-10 are shown as absent with a hyphen.

With this, the first large scale molecular work done to date on *P. davidi*, we now have the information to begin exploring the physiological adaptations of this extraordinary nematode in greater depth.

## Supporting Information

File S1 **The resulting number of contigs of certain lengths resulting from the choice of different kmer sizes on the Illumina data for the transcriptome by Soapdenovo.**
(PDF)

File S2 **A listing of the transcriptome transcripts constructed from the EST and Illumina data with their corresponding match in the nr database at an e-value of 1e-10 or below.** Only those transcripts with a match are represented.
(TXT)

File S3 **SEED subsystem analysis of the two EST libraries PDT (20°C) and PDF (4°C).** The colouring scheme for the legend is read in a counterclockwise manner.
(PDF)

File S4 **Distribution of the genomic scaffold sizes above the N50 value.**
(PDF)

File S5 **Some evidence of heterozygosity in** ***P. davidi*** **.** Pg 1 are the amino acid sequences of a LEA subfamily. Blue: silent substitution, Red: amino acid substitution, Gray italic: missing in LEA1.1 and LEA1.3. Consensus sequences for LEA1 subfamily and general LEA proteins are indicated under the amino acid sequences (shown in blue and magenta, respectively). Pg 2 are the cDNA sequences of the same LEA1 subfamily: Red: single nucleotide variation (SNV) sites. Pg 3 is the trace of the LEA1 gene locus, amplified by PCR from a single worm and directly sequenced from the DNA. The SNV sites found by cDNA analysis (indicated by asterisks and arrow heads) appear as double bands, confirming these sites. Pg 4 are the LEA1 genomic sequences, corresponding to the cDNA sequences. Pg 5 and 6 are cDNA sequences of two other genes, gln-5 and T25B9.9 homologs in *P. davidi*, which also show SNV patterns (the SNV sites are shown in Black/Blue – except for position 628 in the T25B9.9 homolog, which is a sequencing error). Similar SNV patterns have been observed in many other genes including other LEAs, 18s RNA, 28s RNA, and members of HSP-70.
(PDF)

File S6 **Intron size distribution in** ***P. davidi*** **.** The peak around 49 is similar to the intron peak found in *C. elegans.*
(PDF)

File S7 **Cd-hit clustering of the 26 LEA transcripts and the resulting 13 represented amino acid sequences.**
(PDF)

File S8 **Sequences of the HSP-70 and HSP-70-like genes found in** ***P. davidi.***
(PDF)
