# Supplementary material for: Molecular Analysis of the Cold Tolerant Antarctic Nematode, Panagrolaimus davidi
Source: PLoS One. 2014 Aug 6;9(8):e104526. doi: 10.1371/journal.pone.0104526 (PMC4123951; doi:10.1371/journal.pone.0104526)
Supplement: File S1 — The resulting number of contigs of certain lengths resulting from the choice of different kmer sizes on the Illumina data for the transcriptome by Soapdenovo. (PDF) [file pone.0104526.s001.pdf]

Supplementary Table S1 The resulting number of transcripts above certain lengths resulting from the choice of different kmer sizes on the Illumina data for the transcriptome by soapdenovo.

kmer	largest	# > 100	# > 200	# > 500
19	1,465	77,267	7,356	112
21	1,755	118,014	21,350	850
23	1,721	132,829	27,663	1,385
29	2,497	175,320	34,237	2,088
39	2,886	211,691	46,675	3,380
41	3,223	216,531	49,917	3,610
47	3,176	221,660	62,016	5,154
49	3,912	219,760	66,324	5,760
51	4,432	504,589	70,629	6,318
53	3,430	494,214	74,291	6,586
55	3,482	467,647	77,268	6,768
57	3,482	434,161	80,435	7,009
59	3,482	400,984	83,088	7,189
61	3,482	365,864	85,197	7,528
69	3,016	234,165	86,314	7,674
71	3,466	209,703	82,498	7,647
79	3,194	116,842	61,793	6,828
81	2,725	98,050	55,079	6,368
89	2,223	34,594	25,230	3,366
93	1,903	13,085	10,202	1,303
