# Supplementary figures and images for: Molecular Analysis of the Cold Tolerant Antarctic Nematode, Panagrolaimus davidi
Source: PLoS One. 2014 Aug 6;9(8):e104526. doi: 10.1371/journal.pone.0104526 (PMC4123951; doi:10.1371/journal.pone.0104526)

PDT

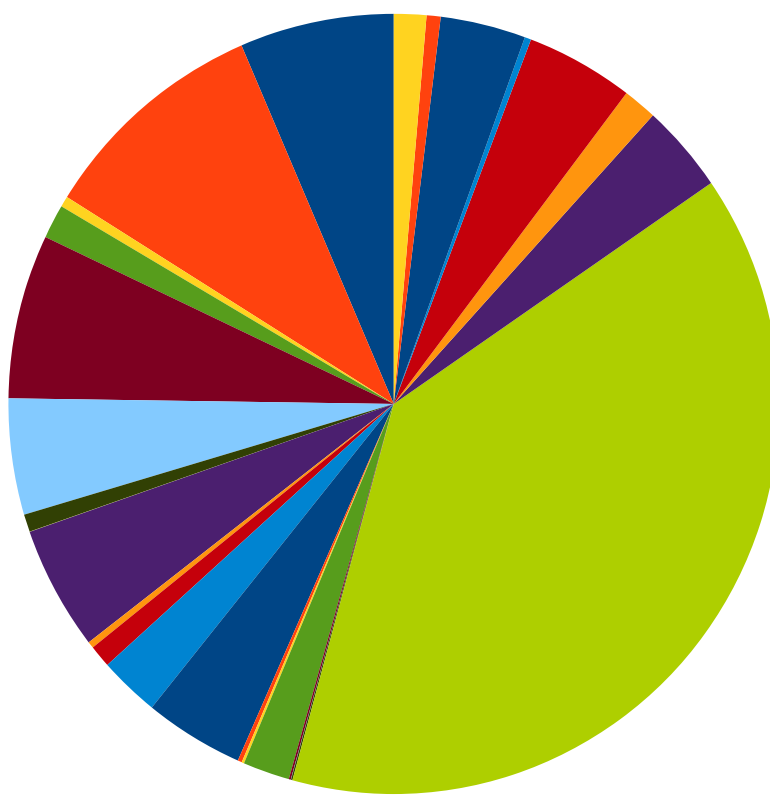

PDF

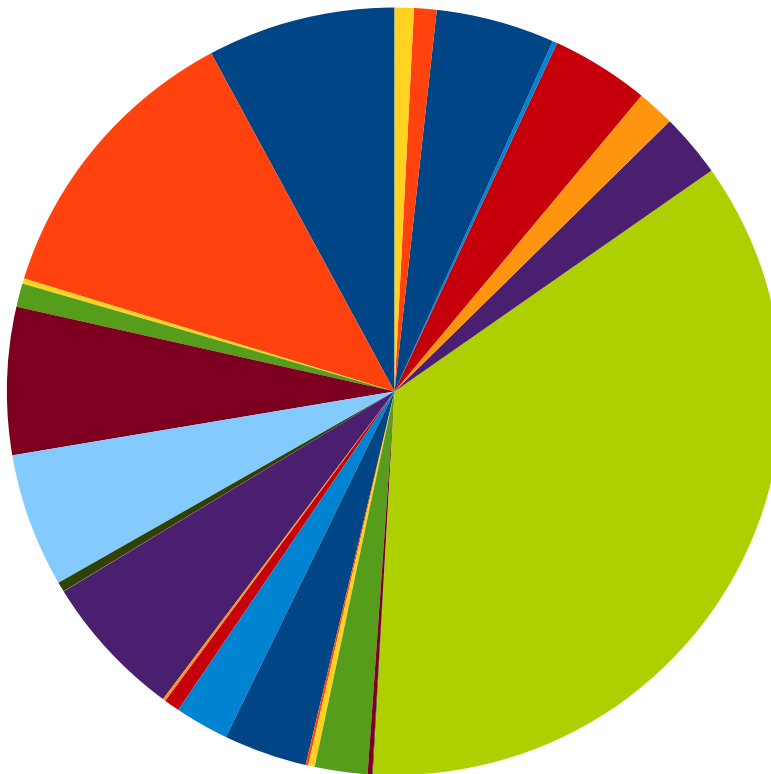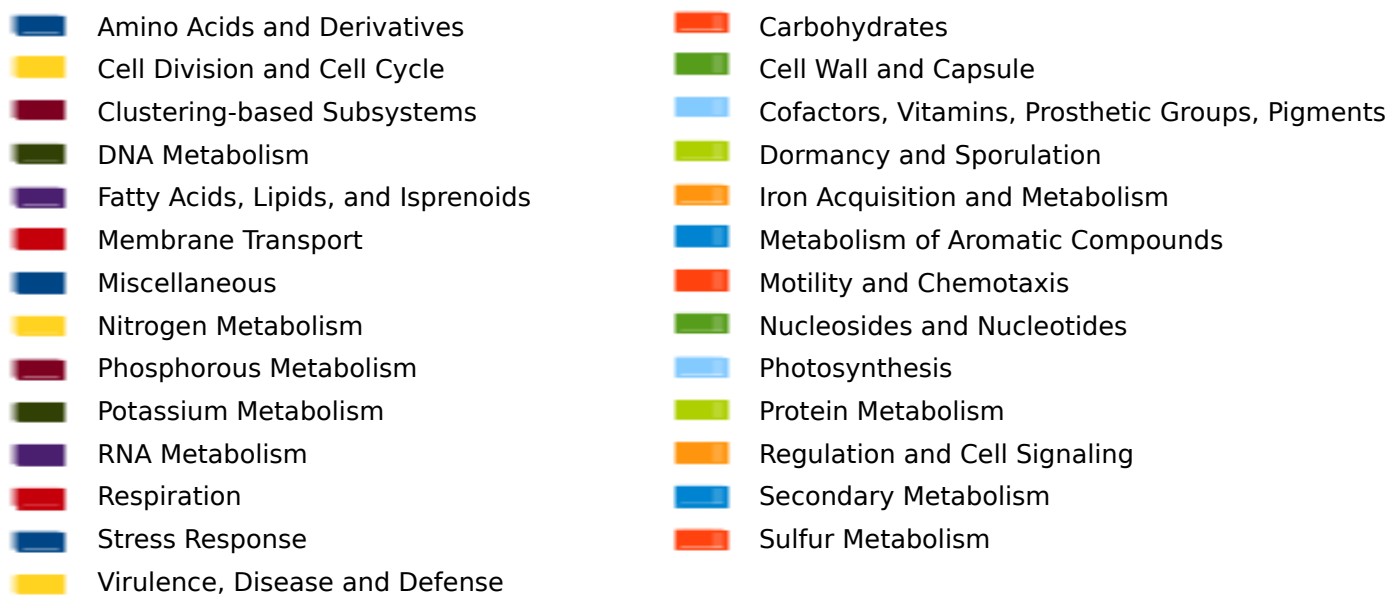

Supplement: File S3 — SEED subsystem analysis of the two EST libraries PDT (20°C) and PDF (4°C). The colouring scheme for the legend is read in a counterclockwise manner. (PDF) [file pone.0104526.s003.pdf]

## Scaffolds larger than the N50

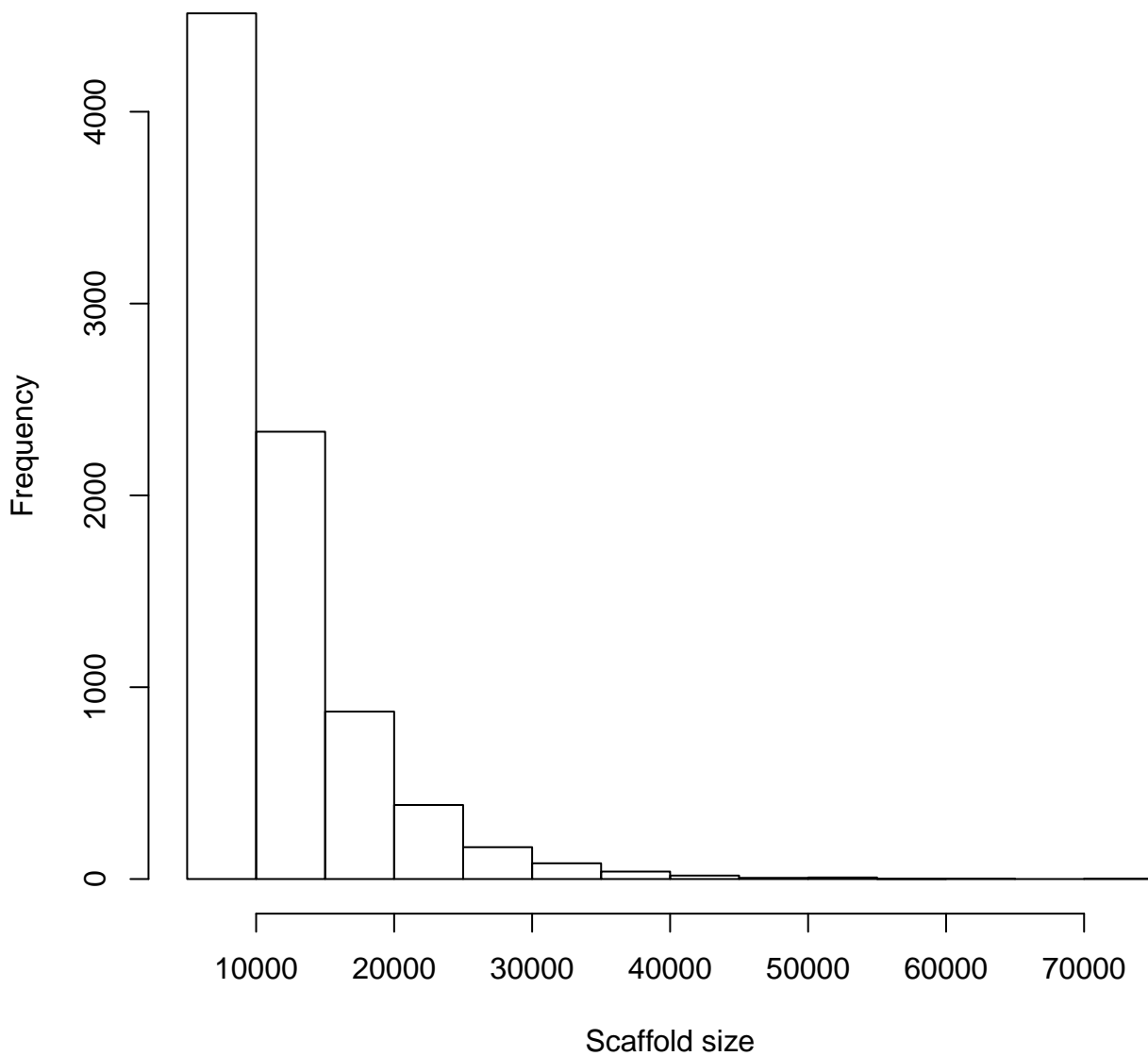

Supplement: File S4 — Distribution of the genomic scaffold sizes above the N50 value. (PDF) [file pone.0104526.s004.pdf]

*P. davidi* intron size distribution

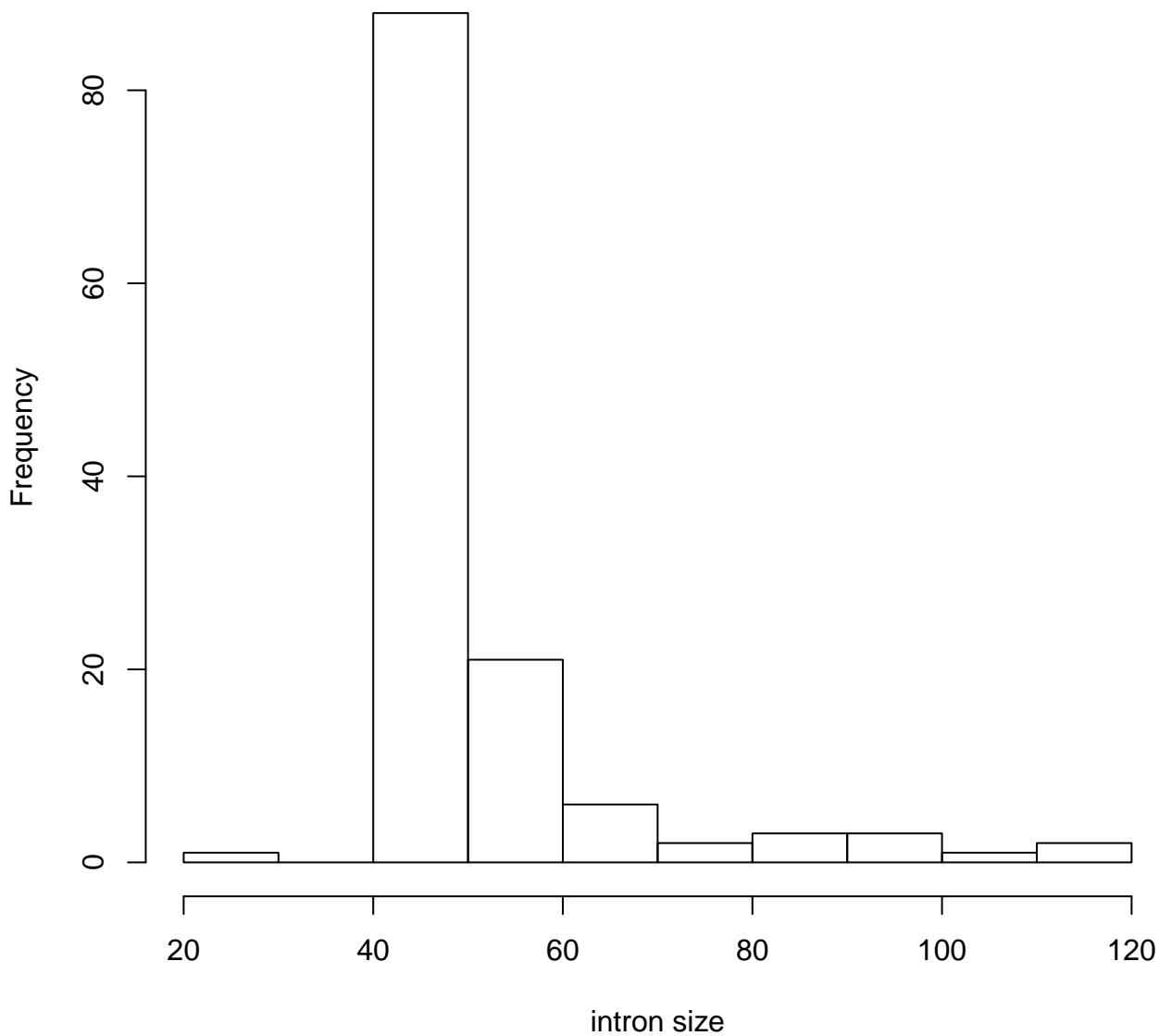

Supplement: File S6 — Intron size distribution in P. davidi . The peak around 49 is similar to the intron peak found in C. elegans. (PDF) [file pone.0104526.s006.pdf]
