# Supplementary material for: Molecular Analysis of the Cold Tolerant Antarctic Nematode, Panagrolaimus davidi
Source: PLoS One. 2014 Aug 6;9(8):e104526. doi: 10.1371/journal.pone.0104526 (PMC4123951; doi:10.1371/journal.pone.0104526)
Supplement: File S5 — Some evidence of heterozygosity in P. davidi . Pg 1 are the amino acid sequences of a LEA subfamily. Blue: silent substitution, Red: amino acid substitution, Gray italic: missing in LEA1.1 and LEA1.3. Consensus sequences for LEA1 subfamily and general LEA proteins are indicated under the amino acid sequences (shown in blue and magenta, respectively). Pg 2 are the cDNA sequences of the same LEA1 subfamily: Red: single nucleotide variation (SNV) sites. Pg 3 is the trace of the LEA1 gene locus, amplified by PCR from a single worm and directly sequenced from the DNA. The SNV sites found by cDNA analysis (indicated by asterisks and arrow heads) appear as double bands, confirming these sites. Pg 4 are the LEA1 genomic sequences, corresponding to the cDNA sequences. Pg 5 and 6 are cDNA sequences of two other genes, gln-5 and T25B9.9 homologs in P. davidi, which also show SNV patterns (the SNV sites are shown in Black/Blue – except for position 628 in the T25B9.9 homolog, which is a sequencing error). Similar SNV patterns have been observed in many other genes including other LEAs, 18s RNA, 28s RNA, and members of HSP-70. (PDF) [file pone.0104526.s005.pdf]

P. davidi LEA-1

LEA-1.1

```
MDSTND
SSPTLAEIAAE
AAHNIAEKAQN
VAAAARDKLAD AYEAVAE
YAHDGKEKAED
LAEAGKEKAED
LGESVKESAQN AAEKVKD
LAHDGKEKVKD
LAQAGKEKAED
LAQAGKEKAQH ASDKAKD
LAHDGKEKAED
LGERAKEKSHD
LAEAGKEKAQR ASEKAKD
VAQSGKEKAHD
LAESGKQKANE
LGDRAKEKADH ASDKAKD
LAQAGKEKAHD
LAQAGKEKAQH ASDKAKD
LAQDGKEKAQE
LGDRSKENAHH ASNKAKE
LANRGNEKAQE
LGDHAKEKASH ASDKAKE
LANRGNAKAQE
LGDRAKEKAES NK*

 AE       QD       D
L DAGKEKA   ASDKAK
 GQ       EE       E

    E    E    E
ØØ XØK KØXD
    Q    Q    Q
```

LEA-1.2

```
MDSTND
SSPTLAEIAAE
AAHNIAEKAQN
VAAAARDKLAD AYEAVAE
YAHDGKEKAED
LAEAGKEKAED
LGASVKESAQN AAEKVKD
LAHDGKEKVKD
LAQAGKEKAED
LAEAGKEKVQH ASDKAKD
LAHDGKEKAED
LGERAKEKSHD
LAEAGKEKAQR ATEKAKD
VAQSGKEKAHD
LAESGKQKANE
LGDRAKEKADH ASDKAKD
LAHAGKEKAHD
LAQAGKEKAQH ASDKAKD
LAQDGKEKAQE
LGDRSKENAHH ASNKAKE
LANRGNEKAQE
LGDHAKEKASH ASDKAKE
LANRGNAKAQE
LGDRAKEKAES NK*

 AE       QD       D
L DAGKEKA   ASDKAK
 GQ       EE       E

    E    E    E
ØØ XØK KØXD
    Q    Q    Q
```

LEA-1.3

```
MDSTND
SSPTLAEIAAE
AAHNIAEKAQN
VAAAARDKLAD AYEAVAE
YAHDGKEKAED
LAEAGKEKAEG
LGESVKESAQN AAEKVKD
LAHDGKEKVKD
LAQAGKEKAED
LAEAGKEKVQH ASDKAKD
LAHDGKEKAED
LGERAKEKSHD
LAEAGKEKAQR ASEKAKD
VAQSGKEKAHD
LAESGKQKANE
LGDRAKEKADH ASDKAKD
LAHAGKEKAHD
LAQAGKEKAQH ASDKAKD
LAQDGKEKAQE
LGDRSKENAHH ASNKAKE
LANRGNEKAQE
LGDHAKEKASH ASDKAKE
LANRGNAKAQE
LGDRAKEKAES NK*

 AE       QD       D
L DAGKEKA   ASDKAK
 GQ       EE       E

    E    E    E
ØØ XØK KØXD
    Q    Q    Q
```

P. davidi lea-1 cDNA sequences

```
        101                                              150
LEA1.1  AAGTGTAAAA GAAAGTGCCC AAAATGCTGC TGAAAAGGTT AAAGATCTTG
LEA1.2  AAGTGTAAAA GAAAGTGCCC AAAATGCTGC TGAAAAGGTT AAAGATCTTG
LEA1.3  AAGTGTAAAA GAAAGTGCCC AAAATGCTGC TGAAAAGGTT AAAGATCTTG
         S  V  K   E  S  A  Q   N  A  A   E  K  V   K  D  L  A

        151                                              200
LEA1.1  CACATGATGG TAAAGAAAAG GTTAAAGATC TTGCTCAAGC TGGAAAAGAA
LEA1.2  CACATGATGG TAAAGAAAAG GTTAAAGATC TTGCACAAGC TGGAAAAGAA
LEA1.3  CACATGATGG TAAAGAAAAG GTTAAAGATC TTGCTCAAGC TGGAAAAGAA
          H  D  G   K  E  K   V  K  D  L   A  Q  A   G  K  E

        201                                              250
LEA1.1  AAAGCCGAAG ATCTTGCTCA AGCTGGAAAG GAAAAAGCCC AACATGCTTC
LEA1.2  AAAGCCGAAG ATCTTGCTGA AGCTGGAAAG GAAAAAGTTC AACATGCTTC
LEA1.3  AAAGCCGAAG ATCTCGCTGA AGCTGGAAAG GAAAAAGTTC AACATGCTTC
        K  A  E  D   L  A  E   A  G  K   E  K  V  Q   H  A  S
                          (Q)                  (A)
        251                                              300
LEA1.1  TGATAAAGCA AAAGATCTTG CACATGATGG TAAAGAAAAG GCTGAAGATC
LEA1.2  TGATAAAGCA AAAGATCTTG CACATGATGG TAAAGAAAAG GCTGAAGATC
LEA1.3  TGATAAAGCA AAAGATCTTG CACATGATGG TAAAGAAAAG GCTGAAGATC
         D  K  A   K  D  L  A   H  D  G   K  E  K   A  E  D  L

        301                                              350
LEA1.1  TTGGAGAACG TGCAAAAGAA AAGTCTCATG ATCTTGCTGA AGCTGGAAAA
LEA1.2  TTGGAGAACG TGCAAAAGAA AAGTCCCATG ATCTTGCTGA AGCTGGAAAA
LEA1.3  TTGGAGAACG TGCAAAAGAA AAGTCTCATG ATCTTGCTGA AGCTGGAAAA
          G  E  R   A  K  E   K  S  H  D   L  A  E   A  G  K
```

P. davidi lea-1 genome sequence

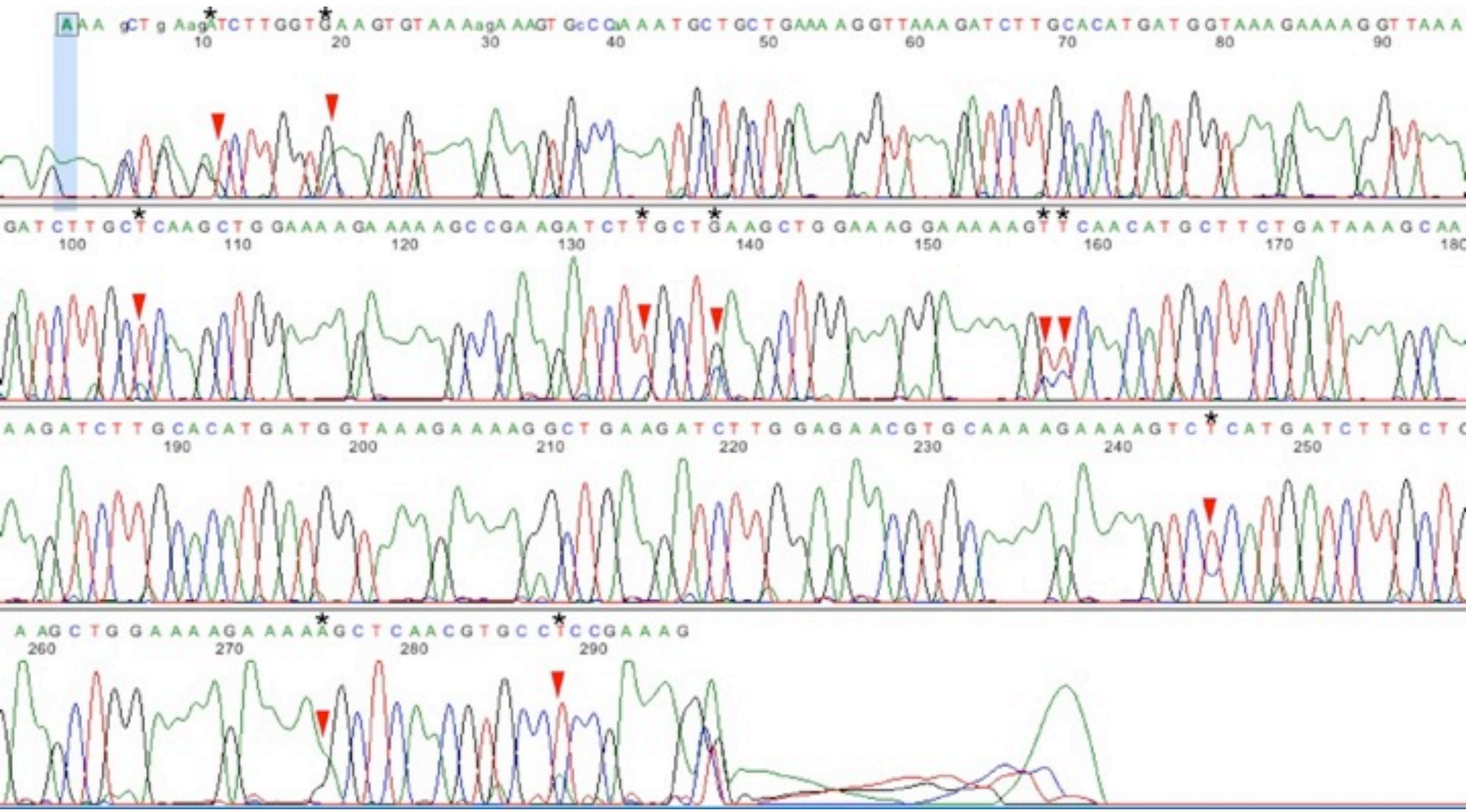

P. davidi lea-1 genome sequences

```
         101                                                150
LEA1.1   AAGTGTAAAA GAAAGTGCCC AAAATGCTGC TGAAAAGGTT AAAGATCTTG
LEA1.2   AAGTGTAAAA GAAAGTGCCC AAAATGCTGC TGAAAAGGTT AAAGATCTTG
LEA1.3   AAGTGTAAAA GAAAGTGCCC AAAATGCTGC TGAAAAGGTT AAAGATCTTG
lea-1F   AAGTGTAAAA GAAAGTGCCC AAAATGCTGC TGAAAAGGTT AAAGATCTTG

         151                                                200
LEA1.1   CACATGATGG TAAAGAAAAG GTTAAAGATC TTGCTCAAGC TGGAAAAGAA
LEA1.2   CACATGATGG TAAAGAAAAG GTTAAAGATC TTGCACAAGC TGGAAAAGAA
LEA1.3   CACATGATGG TAAAGAAAAG GTTAAAGATC TTGCTCAAGC TGGAAAAGAA
lea-1F   CACATGATGG TAAAGAAAAG GTTAAAGATC TTGCWCAAGC TGGAAAAGAA

         201                                                250
LEA1.1   AAAGCCGAAG ATCTTGCTCA AGCTGGAAAG GAAAAAGCCC AACATGCTTC
LEA1.2   AAAGCCGAAG ATCTTGCTGA AGCTGGAAAG GAAAAAGTTC AACATGCTTC
LEA1.3   AAAGCCGAAG ATCTCGCTGA AGCTGGAAAG GAAAAAGTTC AACATGCTTC
lea-1F   AAAGCCGAAG ATCTYGCTSA AGCTGGAAAG GAAAAAGYYC AACATGCTTC

         251                                                300
LEA1.1   TGATAAAGCA AAAGATCTTG CACATGATGG TAAAGAAAAG GCTGAAGATC
LEA1.2   TGATAAAGCA AAAGATCTTG CACATGATGG TAAAGAAAAG GCTGAAGATC
LEA1.3   TGATAAAGCA AAAGATCTTG CACATGATGG TAAAGAAAAG GCTGAAGATC
lea-1F   TGATAAAGCA AAAGATCTTG CACATGATGG TAAAGAAAAG GCTGAAGATC

         301                                                350
LEA1.1   TTGGAGAACG TGCAAAAGAA AAGTCTCATG ATCTTGCTGA AGCTGGAAAA
LEA1.2   TTGGAGAACG TGCAAAAGAA AAGTCCCATG ATCTTGCTGA AGCTGGAAAA
LEA1.3   TTGGAGAACG TGCAAAAGAA AAGTCTCATG ATCTTGCTGA AGCTGGAAAA
lea-1F   TTGGAGAACG TGCAAAAGAA AAGTCYCATG ATCTTGCTGA AGCTGGAAAA
```

cDNA alignment (cluster 257)

gln-5, glutamine synthetase (carbamoyl-phosphate synthetase)

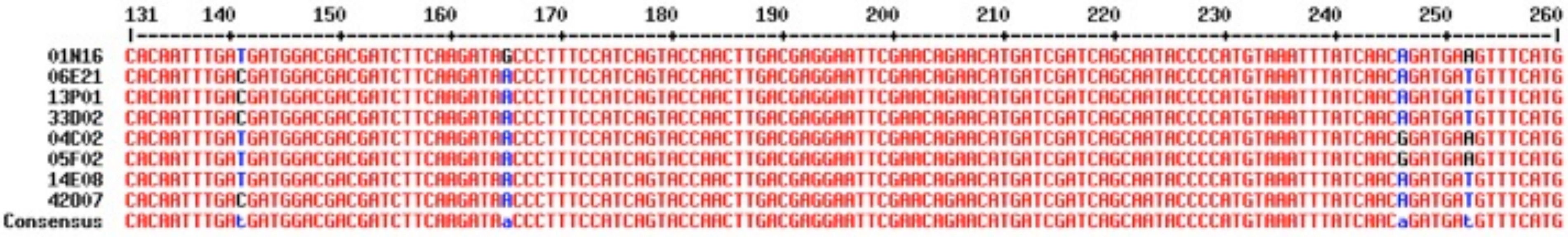

.....

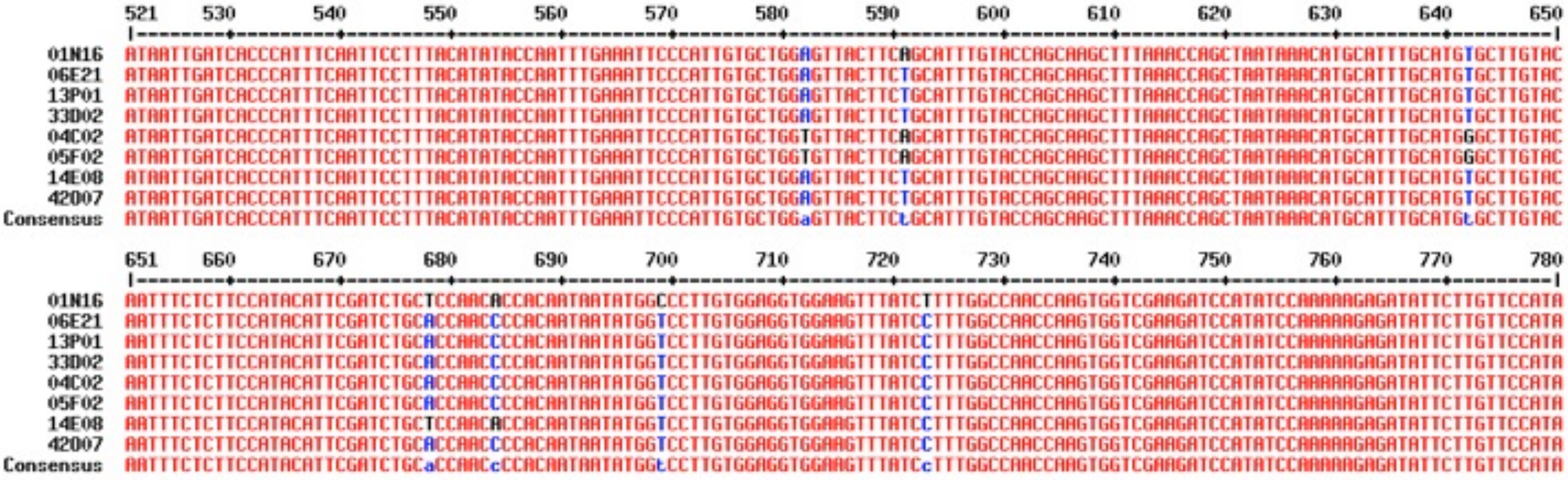

cDNA alignment (cluster 259)

T25B9.9 6-phosphogluconate dehydrogenase

```
          131       140       150       160       170       180       190       200       210       220       230       240       250       260
          |---------+---------+---------+---------+---------+---------+---------+---------+---------+---------+---------+---------+---------|
01N21     TAAGCATTGGATGTAACACGTCCTCCACGACCTGTCCAATTTGTATGAACTGAAGTTTCAGTATCTGTTAAAAGTTTGTAAGTATGTGCTCCAAAATAATCACGTTGAGCTTGTAATAAATTTGCAGGAC
18F09     TAAGCATTGGATGTAACACGTCCTCCACGACCCGTCCAATTTGTATGAACTGAAGTTTCAGTATCTGTTAAAAGTTTGTAAGTATGTGCTCCAAAATAATCACGTTGAGCTTGTAATAAATTTGCAGGAC
23k08     TAAGCATTGGATGTAACACGTCCTCCACGACCCGTCCAATTTGTATGAACTGAAGTTTCAGTATCTGTTAAAAGTTTGTAAGTATGTGCTCCAAAATAATCACGTTGAGCTTGTAATAAATTTGCAGGAC
29C03     TAAGCATTGGATGTAACACGTCCTCCACGACCTGTCCAATTTGTATGAACTGAAGTTTCAGTATCTGTTAAAAGTTTGTAAGTATGTGCTCCAAAATAATCACGTTGAGCTTGTAATAAATTTGCAGGAC
38C13     TAAGCATTGGATGTAACACGTCCTCCACGACCCGTCCAATTTGTATGAACTGAAGTTTCAGTATCTGTTAAAAGTTTGTAAGTATGTGCTCCAAAATAATCACGTTGAGCTTGTAATAAATTTGCAGGAC
28M21     TAAGCATTGGATGTAACACGTCCTCCACGACCTGTCCAATTTGTATGAACTGAAGTTTCAGTATCTGTTAAAAGTTTGTAAGTATGTGCTCCAAAATAATCACGTTGAGCTTGTAATAAATTTGCAGGAC
36L02     TAAGCATTGGATGTAACACGTCCTCCACGACCCGTCCAATTTGTATGAACTGAAGTTTCAGTATCTGTTAAAAGTTTGTAAGTATGTGCTCCAAAATAATCACGTTGAGCTTGTAATAAATTTGCAGGAC
10P19     TAAGCATTGGATGTAACACGTCCTCCACGACCCGTCCAATTTGTATGAACTGAAGTTTCAGTATCTGTTAAAAGTTTGTAAGTATGTGCTCCAAAATAATCACGTTGAGCTTGTAATAAATTTGCAGGAC
Consensus TAAGCATTGGATGTAACACGTCCTCCACGACCcGTCCAATTTGTATGAACTGAAGTTTCAGTATCTGTTAAAAGTTTGTAAGTATGTGCTCCAAAATAATCACGTTGAGCTTGTAATAAATTTGCAGGAC
```

.....

```
          521       530       540       550       560       570       580       590       600       610       620       630       640       650
          |---------+---------+---------+---------+---------+---------+---------+---------+---------+---------+---------+---------+---------|
01N21     CAATCAAATACTCGACTTGCTTCACCCAAAAGCATAAAACCTTGAGCATAACTAACAATTTTTGAAGCATACAATGCTTGTCCCAATTTCAAAATAAAATCTTTTTTTTATTTTTAAATGCTTCTGGTGTT
18F09     CAATCAAATACTCGACTTGCTTCACCCAAAAGCATAAAACCTTGAGCATAACTAACAATTTTTGAAGCATATAATGCTTGTCCCAATTTCAAAATAAAATCTTTTTTT-ATTTTTAAATGCTTCTGGTGTT
23k08     CAATCAAATACTCGACTTGCTTCACCCAAAAGCATAAAACCTTGAGCATAACTAACAATTTTTGAAGCATATAATGCTTGTCCCAATTTCAAAATAAAATCTTTTTTT-ATTTTTAAATGCTTCTGGTGTT
29C03     CAATCAAATACTCGACTTGCTTCACCCAAAAGCATAAAACCTTGAGCATAACTAACAATTTTTGAAGCATACAATGCTTGTCCCAATTTCAAAATAAAATCTTTTTTT-ATTTTTAAATGCTTCTGGTGTT
38C13     CAATCAAATACTCGACTTGCTTCACCCAAAAGCATAAAACCTTGAGCATAACTAACAATTTTTGAAGCATATAATGCTTGTCCCAATTTCAAAATAAAATCTTTTTTT-ATTTTTAAATGCTTCTGGTGTT
28M21     CAATCAAATACTCGACTTGCTTCACCCAAAAGCATAAAACCTTGAGCATAACTAACAATTTTTGAAGCATATAATGCTTGTCCCAATTTCAAAATAAAATCTTTTTTT-ATTTTTAAATGCTTCTGGTGTT
36L02     CAATCAAATACTCGACTTGCTTCACCCAAAAGCATAAAACCTTGAGCATAACTAACAATTTTTGAAGCATATAATGCTTGTCCCAATTTCAAAATAAAATCTTTTTTT-ATTTTTAAATGCTTCTGGTGTT
10P19     CAATCAAATACTCGACTTGCTTCACCCAAAAGCATAAAACCTTGAGCATAACTAACAATTTTTGAAGCATATAATGCTTGTCCCAATTTCAAAATAAAATCTTTTTTT-ATTTTTAAATGCTTCTGGTGTT
Consensus CAATCAAATACTCGACTTGCTTCACCCAAAAGCATAAAACCTTGAGCATAACTAACAATTTTTGAAGCATAtAATGCTTGTCCCAATTTCAAAATAAAATCTTTTTTT.ATTTTTAAATGCTTCTGGTGTT

          651       660       670       680       690       700       710       720       730       740       750       760       770       780
          |---------+---------+---------+---------+---------+---------+---------+---------+---------+---------+---------+---------+---------|
01N21     GGAAGTGGACTTCCTGGAAGAACCTTTGAAGCAGAAACTCTTTGTTCTTTTAAAGCAGATAAACATCGAGCAAAAACAGCTTCAGCAATAAGTGTTACAGGTTGTCCATATTCTAATGCAGAAATACCAG
18F09     GGAAGTGGACTTCCTGGTAGAACCTTTGAAGCAGAAACTCTTTGTTCTTTTAAAGCAGATAAACATCGAGCAAAAACAGCTTCAGCAATAAGTGTTACAGGTTGTCCATATTCTAATGCAGAAATACCAG
23k08     GGAAGTGGACTTCCTGGTAGAACCTTTGAAGCAGAAACTCTTTGTTCTTTTAAAGCAGATAAACATCGAGCAAAAACAGCTTCAGCAATAAGTGTTACAGGTTGTCCATATTCTAATGCAGAAATACCAG
29C03     GGAAGTGGACTTCCTGGAAGAACCTTTGAAGCAGAAACTCTTTGTTCTTTTAAAGCAGATAAACATCGAGCAAAAACAGCTTCAGCAATAAGTGTTACAGGTTGTCCATATTCTAATGCAGAAATACCAG
38C13     GGAAGTGGACTTCCTGGTAGAACCTTTGAAGCAGAAACTCTTTGTTCTTTTAAAGCAGATAAACATCGAGCAAAAACAGCTTCAGCAATAAGTGTTACAGGTTGTCCATATTCTAATGCAGAAATACCAG
28M21     GGAAGTGGACTTCCTGGAAGAACCTTTGAAGCAGAAACTCTTTGTTCTTTTAAAGCAGATAAACATCGAGCAAAAACAGCTTCAGCAATAAGTGTTACAGGTTGTCCATATTCTAATGCAGAAATACCAG
36L02     GGAAGTGGACTTCCTGGTAGAACCTTTGAAGCAGAAACTCTTTGTTCTTTTAAAGCAGATAAACATCGAGCAAAAACAGCTTCAGCAATAAGTGTTACAGGTTGTCCATATTCTAATGCAGAAATACCAG
10P19     GGAAGTGGACTTCCTGGTAGAACCTTTGAAGCAGAAACTCTTTGTTCTTTTAAAGCAGATAAACATCGAGCAAAAACAGCTTCAGCAATAAGTGTTACAGGTTGTCCATATTCTAATGCAGAAATACCAG
Consensus GGAAGTGGACTTCCTGGtAGAACCTTTGAAGCAGAAACTCTTTGTTCTTTTAAAGCAGATAAACATCGAGCAAAAACAGCTTCAGCAATAAGTGTTACAGGTTGTCCATATTCTAATGCAGAAATACCAG
```
