# Supplementary material for: Molecular Analysis of the Cold Tolerant Antarctic Nematode, Panagrolaimus davidi
Source: PLoS One. 2014 Aug 6;9(8):e104526. doi: 10.1371/journal.pone.0104526 (PMC4123951; doi:10.1371/journal.pone.0104526)
Supplement: File S7 — Cd-hit clustering of the 26 LEA transcripts and the resulting 13 represented amino acid sequences. (PDF) [file pone.0104526.s007.pdf]

Cd-hit clusters of the LEA transcripts:

```
>Cluster 0
0	502aa, >PdU002998_v1.1... at 100%
1	593aa, >PdU053999_v1.1... at 82%
2	1002aa, >PDT19L04... *
>Cluster 1
0	339aa, >PdU001599_v1.1... at 99%
1	594aa, >PDT20F06... *
>Cluster 2
0	272aa, >PdU010487_v1.1... at 100%
1	207aa, >PdU017016_v1.1... at 96%
2	593aa, >PDT30I10... *
>Cluster 3
0	320aa, >PDT06A14... *
>Cluster 4
0	307aa, >PdU055885_v1.1... at 89%
1	288aa, >PDF06F09... at 95%
2	317aa, >PDF40F19... *
3	306aa, >PDF16M17... at 99%
4	201aa, >PDF82L15... at 94%
>Cluster 5
0	240aa, >PdU015612_v1.1... at 91%
1	119aa, >PdU017506_v1.1... at 89%
2	282aa, >PDF80K07... *
>Cluster 6
0	235aa, >PdU055084_v1.1... *
1	235aa, >PDT10D20... at 99%
>Cluster 7
0	209aa, >PDF96E03... *
>Cluster 8
0	169aa, >PdU013001_v1.1... *
>Cluster 9
0	150aa, >PdU014555_v1.1... *
>Cluster 10
0	90aa, >PDF39E07... at 82%
1	98aa, >PDT45G14... *
>Cluster 11
0	78aa, >PDF77N04... *
>Cluster 12
0	54aa, >PDT56F11... *
```

Sequences:

>PdU013001_v1.1
MAGITENVKEMLNTVAEKSSEFKDYATGEELRNPSDTSDMISDAKDKASEIADAAKDKASD
LADAASNKASEVKDAASNKASEMKDAASSKASDLSDKASEMKDAAADKASEMYEGAKQ
KASDLSGAAKVKADEMTSQETKDAARDMADKAGSKLEDAGHKIREQKDAI
>PdU055084_v1.1
MSTEKSYTETVQDAITTAREKIIETAEAVKEKAAELATAAGETLGLTSEEAKDKVDDVKDQ
AKEKASEVADAAGKKLDSAKEQVKDKVDETKQKASKAADAAGEKLESAKEHVKDKVGE
AEEKMDDGPELKGNHPPADKMSGGVRIARKKRPSESARHEHGNQEENAASGSADEDEEAP
STTEIAVQKQEKKLQQNFQEDAVKSYHEKPMPTHQPDHQYHSGRAHKDPAFQPKKQ
>PdU014555_v1.1
MPTLYKKVGPLSTTFDSLSMSDNEDKTLGEKAKDAIANAAEAVKEAATDLKNKIVGEKSTE
EKAADKVKDAADAAADKIGDMKDSANNAGDKIKEAATDLKNKVVGEKSTEDKVADKVK
DAADAAADKVGEMKESARDGAHKAGDKLKDA
>PDF40F19
MDSTNDSSPTLAEIAAEAAHNIAEKAQNVAAAARDKLADAYEAVAEYAHDGKEKAEDLAE
AGKEKAEDLGASVKESAQNAAEKVKDLAHDGKEKVKDLAQAGKEKAEDLAEAGKEKVQ
HASDKAKDLAHDGKEKAEDLGERAKEKSHDLAEAGKEKAQRATEKAKDVAQSGKEKAH
DLAESGKQKANELGDRAKEKADHASDKAKDLAHAGKEKAHDLAQAGKEKAQHASDKA
KDLAQDGKEKAQELGDRSKENAHHASNKAKELANRGNEKAQELGDHAKEKASHASDKA
KELANRGNAKAQELGDRAKEKAESNK
>PDF96E03
MLLKRLKILHMMVKKRLKILLKLEKKKPKILLKLERKKPNMLLIKQKILHMMVKKRLKILE
NVQKKSHDLAEAGKEKAQRASEKAKDVAQSGKEKAHDLAESGKQKANELGDRAKEKAD
HASDKAKDLAQAGKEKAHDLAQAGKEKAQHASDKAKDLAQDGKEKAQELGDRSKEKA
SHASDKAKELANRGNAKAQELGDRAKEKAESNK
>PDT06A14
MNRKIITLCFIGLFALSAFADETVGDKVDEGINKASSGVQDAADSVKEKAEEAKQRFRRDT
VGDKLDQGLDSASQSVQNAADSVKDSAKDAKYKVGEALERKRRETVGDKVDEKINDAS
DSVQNAADSVKDKAEDAKQRMKRETVGDKVDEGINKASSGVQDAADSVKEKAEETKQK
RETVGDKVDEKINDAKMTAEKAGESIKETGQELKQDRETVGAKINHGIDAASEKTQDAAE
SVKDTAKDAKYKAGEAIDDAKEGGKTFFENVGETISNAAESVKKAVVNAKDAVVGGSSSS
ESHEEIATTMLIPRYRRHVAYSL
>PDF80K07
MNRKIITLCFIGLFALTAFADETVGDKVDEGINKASSGVQDAADSVKEKAEETKQRFRRDT
VGDKLDQGLDSASQSVQNAADSVKDSAKDAKYKAGEALERKRRETVGNKVDEKINDAS
DSVQNAADSVKEKAEEIKQKRETVGDKVDEKINDAKMTAERAGESIKETGQELKQDRETV
GSKINHGMDAASEKTQDAAESVKDTAKDAKYKAGEAIDDAKEGGKTFFENVGETISNAAE
SVKKAVVNAKDAVVGGSSSSESHEEIATTMLIPRYRRHVAYSL
>PDT19L04
MLFYNANFVQKSWIHEQRTTKMFSKNLIFIAIAIFAITALADETVGDKVDQGINKAAGGVQ
DAAQSVKDTAKDAQQRMKRETVGDKVDEKINAASESVQNAGQAVKDKAEEMKQERKRR
ETVGEKVDAKINAASDAVQNAGEAVKEKGQEMKQERIKRETVGDKVDEKINEASDSVQN
AGQAVKDKAEEMKQERMRRETETIGEKLDSTLNSAGDSIKSAGQSVKEKGQDIKDRKRRE
TVGDKVDEKINAASESVQNAGQAVKDKAEEMKQERKRRETVGEKVDEKINAASDAVQNA
GEAVKEKGQEMKQERMRRETVGDKVDEGINKAADGVQDAAQSVKDTAKDAQQRMKRE
TVGDKVDEKINEASDSVQNAGQAVKDKAEEMKQERLRRETVGDKVDEKINQASDSVQAA
GEKTKEMAKEAKQHKRETETIGEKLDSTLNSAGDSIKSAGQSVKEKGQDIKDRKRRET
VGEKVDEKINAASDAVQNAGEAVKEKGQEMKQERIKRETVGDKVDEKINEASDSVQNAG
QAVKDKAEEMKQERMRRETETIGEKLDSTLNSAGDSIKSAGQSVKEKGQDIKDRKRRETV
GDKVDEKINAASESVQNAGQAVKDKAEEMKQERKRRETVGEKVDEKINAASDAVQNAGE

AVKEKGQEMKQERMRRETVGDKVDEGINKAADGVQDAAQSVKDTAKDAQQRMKRETV
GDKVDEKINEASDSVQNAGQAVKDKAEEMKQERLRRETVGDKVDEKINQASDSVQAAGE
KTKEMAKEAKQHKRETETIGEKLDSTLNSAGDSIKSAGQSVKEKGQDIKDRKRRETVGEK
VDEKINAASDAVQNAGEAVKEKGQEMKQERKRRETVGEKVDEKINEASDSVQNAGQAVK
DKAEEMKQERLRRETVGEKVDEKINAASDAVQEAGEAVKDKGEEMKQDRKRRETVGEK
VDSGVSSARDTMTETGESIKQTGKNMQQDRKRRTILGSDIEQVNDIPVTQITPRHRRYVL
>PDT30I10
MFSKNLIFIAIAIFTITACADETVGDKVDQGINKAAGGVQDAAQSVKDTAKDAQQRMKRET
VGDKVDEKINAASESVQNAGQAVKDKAEEMKQERKRRETVGEKVDEKINAASDAVQNAG
EAVKEKGQEMKQERIKRETVGDKVDEKINEASDSVQNAGQAVKDKAEEMKQERMRRETE
TIGEKLDSTLNSAGDSIKSAGQSVKEKGQDIKDRKRRETVGDKVDAKINAASDAVQNAGQ
AVKDKAEEMKQERMRRETVGDKVDEKINAASDAVQNAGEAVKEKGQEMKQERKRRETV
GDKVDEKINQASDSVQAAGEKTKEMAKEAKQHKRETETIGEKLDSTLNSAGDSIKSAGQS
VKEKGQDIKDRKRRETVGDKVDAKINAASESVQNAGQAVKDKAEEMKQERLRRETVGEK
VDEKINAASDAVQNAGEAVKEKGQEMKQERKRRETVGDKVDEKINAASDAVQNAGEAVK
EKGQEMKQERKRRETVGDKVDEKINAASDAVQEAGEAVKDKGEEMKQDRKRRETVGEK
VDSGVSSARDTMTETGETIKQTGKNMQQDRKRRTILGSDIEQVNDIPVTQITPRHRRYVL
>PDT20F06
MKTIFTFVLLCIGLTLAAETIGQKLDATLDAVSESIGNAADVVKEKTRGSKYTAADAVDKTK
ETIGEAADIAQEYGGMAAEVAKEYGGVAYEKMAEYAAEASEIIRDNAGQFYDNMGKYVK
DAAGNLRDHGGEIAYNAGKYVVYDKTGNLLIDASEYVKDNGGNLLDKSGKIAYKAGEYVI
NQANRVVSNAKDRAQEMASDASDYVKDNAGNLRTKSGEIAYKAGEYVIDKANKVVQNA
KDSVSGTAEDAKNRAQEALEDASQYVKDSMGNLKNKAGEIVYKAGEYAIDQKNKIVRQA
SNAKDDAINRAQEALEDASQYVKDSMGNLRNKKDEIVYKAGEYVVDQKNKIVRQASNAK
DDAKNKAGKYAEDAKYRAQQAAEDASQYVKDKMGNLRDKSNNIVYKVNEYVIDSQNM
VSRKADDAKSRAQELGEDLSQYVKDKKNNYLNKAGEIVYHAGQYVIDEKKNLIRKVSDQ
ADDLGDYVRDAQGNLRDQAGNLIYKAGEQVKRKASQMADQAGDYAEAARDAAGNLME
DAGDKLHSAGKKLKSTDKDDVKDAAHRAGEKLKETGEEVQSGGIYDSIREKVSNAAKTV
REKLDL
>PDF77N04
MSKDNNNPPEKEKYRGFASWPKEKIQEAGKKGGEARKEQLGLKGYIDMGRKGGMARSN
AAAKGDPSTSDKSDKKDGKK
>PDT45G14
MSTEDKSYVEQAKDAAASAAETAKEYAQSAAETATQLATDLKNKVVGEKSTEDKAADKV
KDAADATADKVSDMKDSAKDAAHKAGDKVKEAGNKIQNA
>PDT56F11
MSLKRVLIAEQRFLSTKSTKDSMKDSAASVAENAKEAAKNVAEKLQHAAAAARE
