# Supplementary material for: Molecular Analysis of the Cold Tolerant Antarctic Nematode, Panagrolaimus davidi
Source: PLoS One. 2014 Aug 6;9(8):e104526. doi: 10.1371/journal.pone.0104526 (PMC4123951; doi:10.1371/journal.pone.0104526)
Supplement: File S8 — Sequences of the HSP-70 and HSP-70-like genes found in P. davidi. (PDF) [file pone.0104526.s008.pdf]

Sequence (partial) of HSP-70 and HSP-70-like genes in *P. davidi*

>group-1/5 (HSP-1)
MVNAIGIDLGTTYSCVGVFQNGKVEIIANDQGNRTTPSYVAFTDTERIIGDGAKNQVAMNPHNTVFDAKRLIG
RKFDDAAVQSDMKHWPFKVISADGGRPKVQVEFKGETKEFFPEEISSMVLTKMKETAEAFLGQAVKDAVVTV
PAYFNDSQRQATKDAGAIAGLNVLRIINEPTAAAIAYGLDKKGTGERNVLIFDLGGGTFDVSILTIEDGIFEVKST
AGDTHLGGEDFDNRMVNHFVAEFKRKHKKDVASNPRALRRLRTACERAKRTLSSSTQASIEIDSLFEGIDYYT
SITRARFEELCADLFRNTMDPVEKALRDAKMDKAQIHDIVLVGGSTRIPKVQKLLSDFFSGKELNKSINPDEAV
AYGAAVQAAILSGDKSETVQDLLLLDVAPLSLGIETAGGVMTAIIKRNTTVPTKTSQTFTTYADNQPGVLIQVFE
GERAMTKDNNLLGKFELSGIPPAPRGVPQIEVTFDVDANGILTVSAQDKSTGKKNNITITNDKGRLSKEEIERM
VQEAEKYKGEDEAQRDRVSAKNSLESYCFNMKQTVEDEKVKDKISDDDRKKIVDKCAETLSWLDANQTAEK
EEFEHHLKEIEAVCNPIITKMYQSAGGAPGGFPGGAPGAGGAPGAGGAGGPTIEEVD*
>group-2 (HSP-3/4)
YNDKMRKAGVAVGVSDAGKKDKKYGTGDGTTYSCVGVYKNGRVANDGNRTSYVASATGDRGDAAKNTTN
NTVDAKRGRNDKTVADMKWKVTNKNSKHVNVAVGNDRKASAMVTKMKASYGYKHAVVTVAYNDARATKDA
GTAGNVVRNTAAAAYGDKKDGRNVDGGGTDVSTDNGVVATNGDTHGGDDRVMYKYKKKTGKDRKDHRST
KRRVKAKRASTHVKVVSMDGDSTTRAKNMDRGTKVKVMDADKKDVHVVGGSTRKKDYNGKSRGNDAVAY
GAAVGGVSGDTVDVNTMGTVGGVMTKTRNTVTKKSVSTAADNTVTVGRMTKDNHGKDTGARGVVTDVNG
HVTADKGTGNKNKTTNDNRSDRMNDAKADDKKVKVARNSYAYSKNGDKGGKSDKKTAVDTSWSNKATVDK
HKKDGKVASKYAGTTGGDADAGSDDKD*
>group-3 (HSP-6)
MLSTLRSLASTSRLASRNVYRFKSDIRGHVIGVDLGTTNSCVAVMEGKQAKVIENAEGIRTTPSVVAFTKEGE
RLVGAAAKRQAVTNSQNTLYATKRLIGRRFDDKEVQKDLKVVPYKIVKAQNGDAWVEAQGKVYSPSQVGAY
VLMKMKETAESYLGTPVHNAVVTVPAYFNDSQRQATKDAGQIAGLNVLRVINEPTAAALAYGLDKSDDKIIAVY
DLGGGTFDISILEIQKGVFEVKSTNGDTFLGGEDFDNHLVRFLVDTFKKEQGVDLTKDPMAMQRVREAAEKA
KCELSSTTQTDINLPYITMDASGPKHMALKISRAQFEKLVDDLVKRTIEPCRKALHDAEVKASDIKEVLLVGGM
SRMPKVQELVQQTFGRQPSKAVNPDEAVAMGAAIQGAVLAGNVTDVLLLDVTPLSLGIETLGGVMTKLITRNT
TIPTKKSQVFSTAADGQTQVEIKVYQGERDMAAHNKILGQFSLVGIPPAPRGIPQVEVTFDIDANGIVNVSARD
RGTGKEQQIVIQSSGGLSKDQIENMVREAEKHAAEDAEKKELVETINQAESVVHDTDAKIKEYADQINQEEAK
AIQEKLEALRTKLADKENLKAADVREELSQLQSASLKLFEAAYKKMAEKNSANNADTSSSEPPKEEEKKEEQ
K*
>group-4 (HSP-110-like)
MSIIGVNIGCYSTYVAGIKDGGVEVLANEYSHLPTPTSVSFNSTPRPMGYSSKQQAVPKFKSTCAHFTPLLCR
TVEDHGPILDAIPCQVNAVNLNSDAKDLYSTVTVMDCKNQPITFSIQQVLAAFFTKLFTIAHDATRVHVEECLLA
IPYYLTVADRHVIMEAAQIAGFPFIKLINETAALTASYAFFKKATLPAPPEAPKKVVFVDIGYSQAQIVLSEVHQLG
AKVRYTKHSREVGGFFFDDAIRGYFADEYKKKTKLDARSNRRAWLRLADEAERAKKSVCAASTKTIAQIECL
MEDTDFKAEIDRTLFEELAANVFQAFENLVSSFIAETKINPAEVEVELVGGSSRVPLVRSIVEKHFGHEAKTTM
NQDEAIARGTALMAGLLSPRVRKTAFTLEDFSPFTVFANYYNERREQRSITLVTRGETIPVKKNVTLFIPSVDIV
YEESRTAKRPEFYHIANVVLSRDPEPNPHSVEPTKFRFPFSYDLSHFILNNGCIRIDKELVPEEPKEPAKEEAP
PAKDEKGDASMDQQPPPEKTEKPAKEEAPPAPQGPPQPRTRETRVDHVLNISNGVNPNVNQEYVTQENDM
READAYAIQKAEAKNSLEAKFFDIQRTLEENADTFDATKKENLGQTMLQIEDFLYEDDADYKIEDYSNKLEELK
KLFNEARLPGLQIEEAPANAEAMDVEPQVEEPQ*
>Group6 – no clear homology to *C. elegans*.
MAVAAERLALQNLRPSCNVLGIDLGTTNTVVAIWQAGKPSPEVLTVENNERIIPSYVWCKADGTYDVGRNPV
RNLKQYPQDVCYDAKRMIGQNFTNLTKLQSDYWSFKIVNNNGRPAYELSNGKKIEPEEISVQVLKKAKAIADA
YACANLEYAVITVPAYFDQNQKQATIDAAKRAGLTVLQLETEPTAAAFAFNYDHKRFDDYNLFVFDLGGGTFDI
TIISVKDGRFNVGACGGNNYLGGRDFDNLLMKDLRRRLEEHGAKKFENDVKQRIRLLALAETTKVALTTCDKE
IVNCSDFIANATDKDGMFEITRAEFEKLAFQLKGFLRDACLEAMKRANLTSDEFANVLLIGGSSRMPMVSQLL
NELFPQSFDSIFYQSKGLNPDEAVAYGAAIRAAQLLDPNHRDYKPIENLLPIGIGIGLIENQYSLMIPGGTKYPT
KKKKRYFTTENNQQSASIIVYEGERILASENREILSFVVPNLPPGLAGDVYVDVTFVLDNNGLLTATGKSPTST
AEGSVDYRQVRQAGKPIEQLLLELTLNAENDEQKKILINARVELRKNVENIRNRYENQKNIPLHKRKHIKDSCD
EVIEWLDDNPDEPLDEERINLAKQIEKDAEELIDVRLMTI*
>Group7-N Homology to F44E5.4/F44E5.5
MVFRVGIDPVFGYVSYCNKYEKEIIDIKINNVDYDGIKEVALMFEEIKSKIDGKLGYACIYLSRIYNNEIRKKFIEC
GLKSGFKNVEIFNEETAFYLNAMSQINYKPLNGNVIWIKHFFEIFVWKINNQKAKFCGVWKADRSKLNEFQNIV
DESKLNKGADIVLCNTKIDESNLRKLSECQCFYYNFNFNYWYSKGSLLKARITAGDSELTHLETTCFLTRTFILE
IGNDVITSFEEPQQLPIRYTRTFMKNLKNDTLKVNYTPFELPEYSPIFLTFTINQNGIFSVTFDISK
>Group7-C
EGFQGKPLSKAVITVPAAFNEAQKNATLEAATIAGWKEIILLPEPIAAAFAYFNDRPISNNSNVLLFDLGGGTLD
VCIFKIQNNQIEIISNTGDSKFGGRDFDTVLINYFKNVLSTKYGISFVKHKKYLLMVKCQKIKETLSVMANAGLD
VDDFDANQEGNIEISQEGFQKMCEVLLNKVKNTLNSALHNSNFNANQINKVLHVGGGSRMPMIKQLLRNMFL
EAEHCIEEHPDEVVAIGAAYYAYSLHSDF*

>Group8-N homology to T14G8.3
MVLKPFTSICFSVVLLLVTFCVTHAALAAMSIDFGSQFLKIGLVKPGIPMETVLNKESQRKTANLIAFHNGERYF
GELALQMSYKHPERVIPYVSDLIGKQYNNPIVQDYIKQYPYLNIIEDPVRGTVIINTKDSGSFDIETLVAMILWNA
HEQAVVYGKTPIHDVVITVPSYLNQAERQCIVRATEIAGLTLLQLMSDNGAAALNYAFTRRKEITEKAQNVLIFD
IGASKTTATVVELKLAKSNASKTLEPVVAVKGIGYNRRLGGKILTYKLRDLLAEEFTKKHKTS
>Group8-C
TPDDPKEPESKEEEPKEQQEPETAFNETATNDTVKNQNETVTNGTESSKPKKEQKPKVIKAKLKVKLYKSFV
DLNKENIKESKKILSTFEKVEKLKAERDAAHNGIEARVYELKERLTNEDFTKFAQGNEIENLTKILEETATWIEDE
ADLSTTTEEFKKKRAPIEEIVSAIEKRIRDLIEAELRKKAEAEAKKKAEELAKKKAAEEAKKKEEEALKAAAKEA
EGGEEKPEGTEGGEEKVEGNEDTKEAAGNEEENEKATLDEEETPSVEDILLPKTDDKAHVDL*
>Group9-N Possible group-1 ortholog
VCVEKLCDAITQSIEKEKDVLQRLSNFNIDYEATKSWILEAWSLLSRNENDMNLLAKTLSTINSLIETKEAEINTI
SEKAETFILEGFVEMEDKSNELKIIWEEFIYSLSQKNEKIETIQKFEKQKADISCFATSLIHRLNEVKSLNELQNF
LNDMNQLRDNIISAEMLLNDHEIMKIDNYQEDIQNLYQLHTDLNEKTFNAAKEIFEFEKNDDFSTISNISQLYQY
FDNFNAKHRLSSSKLELKR
>Group9-C
EEIASILLLHMKNKAEEFQGRGISTVVITIPASFTEKQKDATIVGGKLAGWQTINLLPEPVAAAFAYFIDRPLPEN
SNILLFDFGGGTLDICIFQIKNSVFKITRNKGDANIGGRKFDNIIFEYFRNELLLKHEIDIEKNERKKYKLMLQCQ
NVKHNLSIRNDDQIHVDDYDSSKDGPPIMLTRQQFENLTEDLINDIKNLLKSATDDLQHPIEKVLHVGGSSRMP
LIKKILQDLFPHAEHCIQEQPEEVVAIGAAYYSYHLAWKQTQTIQNREELKW
>Group10
DKSFQTFWRNRQSRLQSFKVNNKNGMYDDNWLKRMSKMPKFDIPEKFNSFNTGINAIGIDLGTSSCCVAVN
RENRIEAVAIDITGERTLPSFVGYDEKDVKCGKVVVGNLGMYSKSTIFDTKRVIGKTYAEVEIDPMWLFNVVED
GESVKLEVQGYMNTILRQTPEEVTSELLNYMKQKAEEFQKKAVDNVVITIPASFTDNQKEATLKAAELAGLIHV
HLLPEPVAASITYFIDRRLPNDETILLFDLGGGTLDICIFEVNNFNFKIIRNTGDANIGGRNFDKLLYEYFKEELLS
RHGINVELKDTRKYKLMLKCQEIKHNLSALNDQRICVDDYGPSIDAPPIEITRQNFENMSVDFVNDIKILVKYAT
DNLPNPIAKVLHVGGSSRMPLIKSMLIQMFPNAEQCCEVHPEEVVAVGAAYYSYHLAQQQK*
>Group11-F
TSFVNRFLNLITVNTVNEQKPTFGQEFLNILFIGPTGAGKSMLVNSIYNYLTYNFDEVSTAETVDCILPCHFQLQ
TPNFQTVVFTAGPQDVNEHFNDNGESVTQKPKVYSLKTEKYNCKVIDTPGLGDTRGAKQDEKNVDLIRNAIIE
IEELHAICFVMPSNISKLTSNFEMNMRDLLSLFPKTALKNVFFFFTYANSPFFTTIGDTRSSLEEFISTFEATNNA
KIPFGIENVCCVDSEAFMYFIGTTQGHKYQNRDLESFRISWDKSKAAIENFLTKLEDITPIKSNDILMTYEFEQV
GTLLQLETGKILEKL
>Group11-R
RGEIQQTATEVAAVLLKYMKEKAESFQGKKLSKAVITVPAAFTEAQEAATKKAAIIAGWETVTLLPEPIAASFAYF
IDRPIPNNSILLLFDLGGGTLDVCVFKIISDQIQIISKNGDSKLGGRDFDNLLMDFFSNKLNMDYDVSKLGNNVL
PNLNKDNSETMVNLKDIRDKKFKLLMESQKIKHNLSVHNEDHLDIGDIDPAKTGFIKISQQNFYDLSEDLLNKIQ
NTILSALYKSGYKANEINQVLHVGGGCRMAMIKKLLHEIFSSAKHWCEEHPDEVVAIGAAYYAYEIFSK

The remaining are derived from singleton EST sequences:

>Possible group-1 or group-1 ortholog
MAQDKIGTEKIKSAVITVPDHFLVGQKEATIHAAKLAGIENVKLITEPTAAAFAYGFDHKRFDGYNLFVFDLGGG
TCDVSIVKVEKGEFKVIGHAGDTQLGGRXFDNHLIEYFDKKHKDLRVFESTDEIITKRKMRLREACEALKIALS
HPAKASETIDLDDVAPGTPDEDLTYKQFKEIVANLTRRCRKLCFDALEETELKADDIDEVLLVGGSSKMRVIKD
MLKEVXPTKELSEAINPDEAVAYGATLRAXQXLXPXKXKXXGXXXXLAIGIGVASAE
>PDF16F18F
AKNNFIFFIFQLNLFKKCLMQLELILAHRRRVLEYFKMAKLKLLKMIMAILQLLCMLHSMVWNVMLEKKQKTRF
LQIRKTQFLIFIALLVVNLERMQFNLIQNIGHLRLFLGLMVILKLRWNIKMIQKDFQ
>PDF16F18R
DRTENIYNFLLLDVAPISFGIETAGGVMTPIIKRNTTVPTKTSVTFTTSVDNQPRVVLQVYEGERVMTKDNNLL
GSFELCGIRPAPRGVPTIEVTFNLYTYDHLDVTAQDKLTGKSEILPITKLLLSERKKIYVSVDPDDPNLPALPWID
PEDDITKP*
>PDF20O10F
MMVIHVGIDGLNGYIAAYNDITKEIIEFETAEVDHRDKSINKVHKICRKIEATFLEEFGYACIYIPEYFPKEYRREFI
NNGLQIGFKKVFITGKLVNIYLECFINSEEKPSTGDIFWIFDRNKLVIWEKTERKSEIIQFGPNIFKDDITESDLLF
YKLDSGIDKNPNIIFLDSNHYQDCSETVKTIFPDCKYISHNSILSYCKSALLKARIAADDQDLDAEIKNCDINNFT
ENKIEIWIFXPSGNFDKDPILYLDAGQPLPFEKXMIIPVESXQKI
>PDF20O10R
PIPPNFNLLLFDLGGGTLDLCVFKAEKNKLKVIANNGDSNLGGSDFDMVLVQHFEKILETRYKIIMNEKNRYRLI
QKCLDIKHTLSTESEASLDVSEINFEIDEFLTVTRQEFEQMASKLLDQIGDVLKQTFSKTDIFACDINKVLFVGG
GCRMPMIQHFLRKRFPKAEXSCDENPDGMVAIGAAFYSSFLMSKNDSSNCNIT*

>PDF26D07
VTVFCFWLLYLILGMYGEDLFISFCDKKQKYGFAAAEDYRKNPNFVVFDLIKIMSMLPNSKIEADPSWGFTFTE
NAENPLLIQFDNFDGNKKAASPAFLMAMLLKHHLKIIKSEIGEKPKELGFWLLDKFKADEKERIKNGIKDACTLL
KVSFVEVNV
>PDF40A16 Possible group-10 or group-10 ortholog
KFEKGIPYFKEIEEFDKISCEVKHFLNGFSIEEKFDNEMKKQESLAEIDCKIEQLVQIAKEVTEKEKNLAEYFYM
NLHGIKEMYQLCVENDDEKDEKSVEEKPSLLPIPSTIKLVSSAPNSTNEKYLIPKKLKLYDDKINAVGIDLGTSR
CCTAVNRNNKMETVALDNNGERPLPSYVSFDEKDEKCGKIVVDGLLKNLEKFTCFDIKRIIGKKYSNIIYDKSW
PFTVIEKNGKIFVKTANSHGEITKTPEEISAVLLKYIKEKVSTFQGKILTEAVLTVPAAFTEKQKEATLKAAKLAG
WEKIHLLPEPVAASFAYFNNREIPNNSNVLLFDLGGGTLDVCLFHINDGSLKIIKSNGNTCLGGRDIDNLMMRH
FTTALKFKYNINVNKKVKKKYQLLIECQNIKHNLSVRESDLLDPEKFDSGIEGGSIEIERCELEEMMTELIQRMK
LIIIETLENAKMDHNEVNKVLLVGGGCRMPIVKTMLKNMFPNADQCCEEQPDEVVAVGAAIYAYHLATTNERQ
SEKVEIKKLL
>PDF40K18
TKMFSVLRLLAKSPIRLSQRNVEDIYPCAVTDHVIGVDLGTTNTRVAVMKNGRIKVIENEIGLRKTPSVVAFTEA
GGRLIGAEAKRQAGSNPENTINGVKRIIGRKFGDVQKDLIDVPYKIVKAENGDAWIESRGIGYSPSQITAYLLM
KMKEAAESYLGDYVHHAVITVPPYYSESQREAMYDAGKIAGLKVLRVTSEPGAALFAYGLVKGAREHSSKSE
NGIFAIYDFGGGKFNISIYEMQNGIGICESIFTDKTLGGEEFDNRLLCFLVDTFKMEEDIDLSDYPEAMQRVREA
AEKAKCDLSSVTETCINLPNIIIDASGFKHMIVKISREQFENLVDDLLERTIKLSEKAVVAVGFSSVKEILFIGGMS
RMPKIQELLKKHFGRQPKKGINPEEVVVRGAAFHGAALSGKVPEFARH*
>PDF43N17N
MYNYTASKASDDDIIDGELYYEKRRIQMLRDEYIAIQKKTFTKWINSYLVRGGQDEISDLFVDIRDGTMLLKFLE
IIYSTKLQRPSPKKTRIHCCANINISLEFLRIRGIYYENLSSEDIFDGKPTLILGLIWTIILRSVIEIPKEKSIDQKTIPS
ATEIVLIWCQKMTADYEHVNIKNFTTSWRDGLAFNALIHAHRPELIEYDSLLQSNTIYNLQNALNVAEKYLGISK
LFDAEDIATECPDNKLIFTYVSTYYNYF
>PDF43N17R
KNLNTAVITVPSEFSDKQKEATKMSAEYAGWQNIHFIPEPLAAAYAYFSEIDMPENANILICDCGGGTVDICVGT
VTTNELYVLCYVGDSHLGGRDFDNVLFTHFNGILLHKYKIDVMQSSKKYVLRQKCQNIKHQLSAADKDWLDV
DDFDCSINEVIQITKLEFEEMTTPLLVRIEEVIRRAVAQSNIATTDINYVFQVGGGCRMPMLKKLLLDTFPAANH
QSSLYPDWIVAHGAALYAYYLKNNVAQNHSRKFLEW*
>PDF52M24
QKQITIKAAKMAEWSPRLLPEPIAAAFAYFNNHEYEENSTLLLFDLGGGTLDICLFKVINNSISIINQSGDSFLGG
RDFDNLLFNYFYDRLEHEFGIIVRNDEKKKYKLLAECQKIKQYLSVRNEERLDVDEFAPDNGNTISITRKMFEE
RAKPLLFKIRTFITTILKESGYQSSQISKILLVGGGCRMPMIKELLKNKFPDAKQCCEEQPEEVVAVGAAMYAYH
LMTTK
>PDF57M16F Possible group-1 or group-1 ortholog
GKEKKSVSALSHDKSGILPNFVLKLLFSSLLSVAENIEKIHRMRFKFILLGDCASGKSSLLHRWIFNRFPERCLS
TIGANFMNKEIDIYNKSVSLQIWDTASQERFKSSIAPFCRGVQCCILVFNLAVNDWGDSSIETLDEWLEFFRYY
ETQSPVVLIGNKVDKVDKESRSVSKKEVNLWCSKNNIKAYFEVSAKDGTNVDETLEEIAEIAYNHSQANQYYY
LNTGNEYYLSVFEKEDNLMFNNLKKNFSDGLFNLENMQYHFPKSRTLIMDFLMLLE
>PDF57M16R* Possible group-1 or group-1 ortholog
LIQKGYWKVYKSIDADKSWQFSITHYFNRAKIGIISSDGETYKFPEEISAALLKHVKIKCEEIQGKELNETVITVP
AMFDEIQNEATYAAALLAGWKEIHLLPEPVAAAFAYFIDKPIPSNSKLFLFDLGGGTLDICIFKITGEKLEIIGRSG
DPYLGGRDFDNILIEYFREQLLSKYGVTVAENKKYKLMKECQEIKHNLSLRDDDKLAVEDYDLTKENDYIPITRI
QFETLAKNLLMLIKSNITAALENAGVKSDEINKVLYVGGGSRMPIIKDLLLHETFPNAEHCCEQNPDEVVAVGA
AYYSYHLPSFKTNENRCSVILNLIYA
>PDF75P18F
MSNKTGIFAGIDLGNENSCIAIYKYEKTEVLAGADGSRITPSYVYIGENGEKHVGKGAKNYGHKKPERLFYDIK
RHLTMRSEGSSYKKTQWSIIDEAERKFGYFDYYIDGKFYTPVMILAEIMKKLLSYVGEDVEGVVLTVPAFFSTE
QKNKILKAAEMAGITVLQLICEPTAAAIAYGMEHSYTNGKCYLFLTLEMDLSIYQLSKWLTKLTLKLLFMVVILI
>PDF75P18R
NFKEMVFSYPFNFNEREKEALKTAAAIAGFEDVTLLPDPISAFYAYYMNGPNFNNQTLLVFDLGGSSLELCVL
KVNDKKIDILQTCSSLNVGGKYFDTVLENYLKEFLKLKEKTEPYNLNIEKKQHRLKVDSTTIKEIFEYDTEYEYS
FDNVVTASEEKIPITREKFENLSRDLLLEIQNTVNRTKHWKIDKVLQVGGGCRMSMIKRLLQTEFPNAQHLCH
EFPEEVVAIGAAFYANNLLNATKYEMTIPQHNA*
>PDF82N23
MSTSVSLIGGGILVLLIAGYLAQRNNLIPPPTPKIVGIDLGTTFSSIGVYQAVTGETEILPDSLGKRSIPSVVGFLK
NGTVLIGTRAVEQQEHNPKNTIYDAKRFIGKTFKSDNPQLMSDLKRYPFVVKLDSKGKAYFEVELDSGIRKVT
PEEIGSLIIKYLKEIANEKYQTTIPQLVISVPAEFDENQRNYTKKAAELAGLEVRRIVSEPTAAALAYGLHKKKGV
EYIVVFDLGGGTLDVSIIWLQGGVFVTQAMAGNNRLGGQDFNERTQKHLIQVIEKTFNKNIVDKEDIQQLRLAI
EEAKIRLTDVPVTNIRLQLRTVGEFNYQLTRNEFEELNKDLFDSIIDPIEAALADCNLKREDIDEIVLVGGSTRIPK
VRQVVGSYFGKAPNFGIDPELAVVTGAAVQAGVIGGGWPLEVTAMELPTNRRKVHLYTHNSEA

>PDF88D19
REACEALKIALSHPAKASETIDLDDVAPGTPDEDLTYKQFKEIVANLTRRCRKLCFDALEETELKADDIDEVLLV
GGSSKMRVIKDMLKEVFPTKELSEAINPDEAVAYGATLRAAQLLDPEKSKGFGIVDSLAIGIGVALAENRYHFLI
KRNTPIPTKFGPETYSTVYNNQTAMTFNIYEGERLLADRNHFIMGFELQGLPKKRAEEVKATLELELDGNGILK
AIAKGDGKEATQQVNHNEKKATGKKIEDMLADMEINCDRDREKDRTNAARMEIIKTSI
>PDF90H19
LNAIGIDLGTSECCAFVIRRNGPEGVVLDTVTNQRNMPSYVAINEMNLPCGQNVLNRMVTKSEYSAFDAKFL
MGKELEEIVIDPLWPFEVISYCEEIYITFGDKSFPWIRNSVDISSCLLCHIKTKAEEFQGRMLEEAVITVPSSFTE
KQKDATIDAATYNSTWRLVKLLPEPVAALFAYFYETEIPNGSTVFLFDCGGGTTDICIAKIIGNKINILSEKGDPLF
GGKNFDRILISYFNSVLNHKYGLNVLETNNKYRLMVKCQEIKHTLSVQTDADLFVDDFKTDENDVIPITREEFE
CMAADLICHAKKLIVESLSKAGINPDEINIVFQAGGGCRMQMIKQMLRDMFPKAQHQCSIHLEELVAKGAALY
SYELKIERKIKERMFGK
>PDF96I08 possily group-1 or group-1 ortholog
MMNKPKIIGIDLGTTFSCVGVFEYGKCEIIANGQGERTTPSVVGFIDGERPVGEYPKKKMYKYPSNVISCVKR
LIGRKFDDPNVQKDIPNLSYKVVSKAGNAAIEIELNGKPKIYTPEEISAMILLDLKRTATKYLGCQMDDTIGAVITI
PAYFNDSQRQATIDAAKIAGLNVLRTINEPTAAALAYGYRQKWKEGIILVYNLGGGTFDVSIIKVTNGCCEVLAV
DGDDHLGGEDFDNILVKYCIEEFRRKHGHDISNNRRAICRLKAVCEAAKRLLCNCSHAPIEVENLFGGDDLNC
KITVARFNQLSKELIEKTVELVKKVLEKAKLSKNDITNVLLVGGSTRIPLIEKMLKEFFDQKNLNFDINPDEAVAN
GVTILAAHLSSSCFDTSVQNIKLLDVIPRSLGIRIEIDSIKNIFSVVIKRCTRFPCEITKGFAAFYGQTNADIDVYE
GEDPVSDRNRLLGSFVLRNISPAPIGRPKADVIFKIDENCILTVTAVDQQNGNKDSIEILPDKGRLTESQIFEMIH
EICPPPMEIDIEDD*
>PDF98M19F
MALTSAENLERFKLNPTGPAIGIDLGTTNSCVGYFVNNNVEIITTAAGKRTVPSYVFYNENEGCIIGEQAIARLK
TNPNHVVYDSKRMIGLNYDDNVVNRNKSFWTFTVTKSDNNRPIYDIDKNHQVLPEEVSSEILKELIKMAQDKI
GTEKIKSAVITVPDHFLVGQKEATIHAAKLAGIENVKLITEPTAAAFAYGFDHKRFDGYNLFVFDLGGGTCDVSI
VKVEKGEFKVIGHAGDTQLGGRDFDNHLIEYFDKKHKDLRVFESTDEIITKRKMRLREACEALK
>PDF98M19R
FNKTMPKAMFRCLEETELKADDIDEVLLVGGSSKMRVIKDMLKEVFPTKELSEAINPDEAVAYGATLRAAQLLD
PEKSKGFGIVDSLAIGIGVALAENRYHFLIKRNTPIPTKFGPETYSTVYNNQTAMTFNIYEGERLLADRNHFIMG
FELQGLPKKRAEEVKATLELELDGNGILKAIAKGDGKEATQQVNHNEKKATGKKIEDMLADMEINCDRDREKD
RTNAARMEIIKNINFIKWDCEEKKKKKPRAKELDGLLKECKEVEKWMSSNNAALTPEIREQFEKIEKDAKRLLR
SS*
